# Molecular dynamics simulations reveal the importance of amyloid-beta oligomer *β*-sheet edge conformations in membrane permeabilization

**DOI:** 10.1016/j.jbc.2023.103034

**Published:** 2023-02-16

**Authors:** Dirk Matthes, Bert L. de Groot

**Affiliations:** Computational Biomolecular Dynamics Group, Department of Theoretical and Computational Biophysics, Max Planck Institute for Multidisciplinary Sciences, Göttingen, Germany

**Keywords:** amyloid-beta, Alzheimer disease, ion channel, membrane bilayer, molecular dynamics, oligomerization, peptide conformation, permeability

## Abstract

Oligomeric aggregates of the amyloid-beta peptide(1-42) (A*β*42) are regarded as a primary cause of cytotoxicity related to membrane damage in Alzheimer’s disease. However, a dynamical and structural characterization of pore-forming A*β*42 oligomers at atomic detail has not been feasible. Here, we used A*β*42 oligomer structures previously determined in a membrane-mimicking environment as putative model systems to study the pore formation process in phospholipid bilayers with all-atom molecular dynamics simulations. Multiple A*β*42 oligomer sizes, conformations, and N-terminally truncated isoforms were investigated on the multi-*μ*s time scale. We found that pore formation and ion permeation occur *via* edge conductivity and exclusively for *β*-sandwich structures that feature exposed side-by-side *β*-strand pairs formed by residues 9 to 21 of A*β*42. The extent of pore formation and ion permeation depends on the insertion depth of hydrophilic residues 13 to 16 (HHQK domain) and thus on subtle differences in the overall stability, orientation, and conformation of the aggregates in the membrane. Additionally, we determined that backbone carbonyl and polar side-chain atoms from the edge strands directly contribute to the coordination sphere of the permeating ions. Furthermore, point mutations that alter the number of favorable side-chain contacts correlate with the ability of the A*β*42 oligomer models to facilitate ion permeation in the bilayer center. Our findings suggest that membrane-inserted, layered *β*-sheet edges are a key structural motif in pore-forming A*β*42 oligomers independent of their size and play a pivotal role in aggregate-induced membrane permeabilization.

The progression of Alzheimer’s disease (AD) is marked by synaptic dysfunction, inflammatory processes, and neuronal loss that eventually result in irreversible neurodegeneration and dementia (1, 2). The abnormal accumulation of amyloid-beta peptide(1-42) (A*β*42) is linked *via* several proposed pathways to the observed proteostatic stress and dyshomeostasis in the brain of AD patients (3, 4, 5, 6). A*β*42 self-assembly into soluble neurotoxic oligomeric aggregates, A*β*42 interactions with binding receptors, or downstream accumulation of reactive oxygen species represent some of the potential targets in a diverse set of therapeutic intervention strategies for AD (7, 8). Soluble A*β* oligomers and insoluble fibrillar amyloid plaques do not show the same correlation with cognitive impairment and build-up in AD brain (8, 9), as small A*β* assemblies are suggested to elicit potent neurotoxic activity in the early stages of AD (9). Yet to date, a full understanding of the complex molecular processes has not been achieved. The causal involvement of A*β*42 in the human neuropathology *via* a linear cascade of events therefore remains disputed despite decades of intense research (10). Along these lines, analysis of human cerebrospinal fluid showed that oligomeric A*β* aggregate sizes and structures are heterogeneous and their mechanisms of toxicity vary during the disease progression (11). The direct interaction with cellular membranes and detrimental effects on cell viability caused by oligomeric A*β* aggregates as small as dimers (12, 13) are, however, well established *in vitro* (2, 14, 15, 16, 17, 18). In fact, smaller aggregates were identified as the most potent agents at disrupting membrane integrity (19, 20, 21), suggesting that membrane-associated A*β* oligomers are an important piece to the puzzle. The ability of low molecular-weight A*β*42 oligomers to permeabilize membranes, including a variety of N-terminally truncated A*β*42 variants (22, 23), has sparked the quest to explore the putative presence of ion channel-like (24, 25, 26) and amyloid pore structures (23, 27, 28, 29, 30). Several studies have demonstrated that A*β*42 induces permeation of common physiological ions across planar lipid bilayers and plasma membranes (24, 29, 31, 32, 33), with a preferential flow of calcium and potassium over sodium ions (24).

Still, the inherent heterogeneity and transient nature of the conformational states populated during aggregate formation has thwarted the elucidation of high-resolution structures in general (34) and of pore-forming oligomers in particular (35). Recent advances in functional and structural characterization of small A*β*42 oligomers in membrane mimics therefore critically contribute to the understanding of the molecular basis of pore formation in a near physiological environment (20, 29, 30, 33). Ciudad *et. al* (33) studied preparations of *β*-sheet pore-forming oligomers (*β*PFOs) with solid-state NMR spectroscopy and proposed a membrane-inserted aggregate structure composed of four A*β*42 peptide chains (PDB ID: 6RHY). The structural model exhibits a six-stranded, antiparallel *β*-sheet with two distinct subunits. Two antiparallel *β*-strands from the A*β*42 C-terminus (*β*3, residues G29-I41) make up the aggregate core, with two flanking *β*-hairpins, each formed by residues G9-A21 (*β*1) and G29-V40 (*β*2). Additionally, high-order A*β*42 oligomers, in particular octameric structures, are observed. In both tetramer and octamer structures, exposed *β*-strands constitute the edge of the A*β*42 *β*PFOs transmembrane domain (TMD) (33). The N-terminal edge *β*-strand harbors residues 11 to 16 that have previously been implicated in the formation of stable intramembrane A*β* oligomers (14, 18). Most of the previous A*β*42 *β*PFO structure models were predominantly characterized by *β*-barrel or cylindrin-like conformations that avoid exposed edge strands by continuous intermolecular hydrogen bonding (29, 30, 36, 37) as an important difference to *β*PFOs with the 6RHY fold. Covalently stabilized oligomer conformations from A*β* fragments form nonfibrillar *β*-sandwich structures, however, exclusively with *β*-turn-*β* folds (34, 38). According to Ciudad *et al*., membrane-inserted A*β*42 oligomers with the 6RHY fold expose hydrophilic edge strands that are able to facilitate water permeation and lipid head group perturbation by a mechanism termed edge conductivity (33). Due to their small size, the novel *β*PFOs with the 6RHY fold are suitable model systems to study the oligomer-induced disruption of a model membrane by means of all-atom molecular dynamics (MD) simulations. Here, we address the question if pore formation and ion permeation are common characteristics of many low molecular-weight A*β* oligomer structures or only associated with certain conformational states. We probe the principal relationship between the three-dimensional A*β*42 oligomer structure and membrane permeabilization for more than a millisecond in total simulation time. We monitor the stability of the aggregates and reveal the molecular determinants that render A*β*42 *β*PFOs with the 6RHY fold capable of ion permeation.

## Results

### Extent of membrane permeabilization induced by A*β*42 pore-forming oligomers examined on the *μ*s time scale

**Figure 1:**
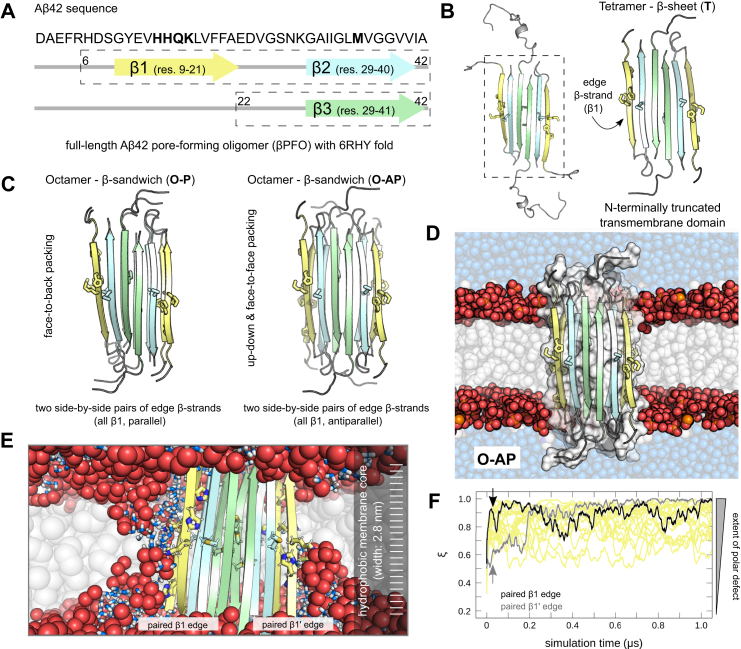
**Simulation model of A*β*42 pore-forming oligomers with the 6RHY fold.***A*, amino acid sequence of full-length A*β*42 is shown together with the transmembrane domain of A*β*42 pore-forming oligomer models with the 6RHY fold highlighted by *boxes* with *broken lines*. *B*, renderings of corresponding full-length A*β*42 tetramer *β*-sheet and its N-terminally truncated transmembrane domain model (T). Structures are shown in cartoon representation, side-chain atoms of residues 13 to 16 (HHQK) and 35 (M) are indicated as *sticks*. *C*, initial coordinates for two octameric *β*-sandwich oligomer models: O-AP and O-P. *D*, snapshot of membrane-embedded A*β*42 O-AP model. *E*, close up view after 10 nanoseconds of MD simulation shows the formation of polar defects along either edge of the A*β*42 oligomer model. Phospholipid atoms from the head group region are shown as *spheres* and highlighted by color (*red* - oxygen, *orange* - phosphorus). Water molecules inside the lipid bilayer are shown as *sticks* (*blue*). *White bars* on the right indicate 28 slices along the membrane normal used to monitor the presence of water and polar lipid atoms across the membrane. *F*, time traces show spontaneous formation of a continuous and stable polar defect expressed as reaction coordinate *ξ*. *Arrows* indicate corresponding *ξ* values for polar defects along the two *β*1-strand pair edges shown in (*E*). MD, molecular dynamics.

Simulation models of pore-forming *β*-sheet oligomers with the 6RHY fold are based on the A*β*42 *β*PFO structure reported by Ciudad *et al*. (33) (Fig. 1*A*). The disordered, solvent-exposed N-terminal regions could not be assigned or did not show lipid interactions experimentally (33). We therefore built a *β*-sheet model (**T**) confining the A*β*(1-42) molecules to two subunits of the transmembrane motif with residues 6 to 42 and 22 to 42, respectively (Fig. 1, *A* and *B*, see Experimental procedures). In addition, a tetramer with full-length A*β*(1-42) was set up (Fig. 1*B*). Two octameric *β*-sandwich models were obtained by packing the hydrophobic core of two N-terminally truncated transmembrane *β*-sheet tetramers side-by-side. We considered only two sterically favorable packing modes of the tetrameric *β*-sheet subunits: up-down and face-to-face (**O-AP**), as well as face-to-back (**O-P**, Fig. 1*C*).

The full-length and N-terminally truncated (**T**) tetramer *β*-sheet as well as the octamer A*β*42 *β*-sandwich oligomer (**O-AP** and **O-P**) models were embedded in a lipid bilayer consisting of zwitterionic 1-palmitoyl-2-oleoyl-sn-glycero-3-phosphocholine (POPC, Figs. 1*D* and S1).

For each of the N-terminally truncated transmebrane domain structure models, 10 independent 2.5 *μ*s long MD simulations were carried out with two different MD force fields (AMBER99SB^∗^/Slipids referred to as AMBER and CHARMM36m/CHARMM36 lipid force field referred to as CHARMM, see Experimental procedures). A detailed summary of all simulated systems and performed MD simulations is provided in Tables S1–S3.

**Figure 2:**
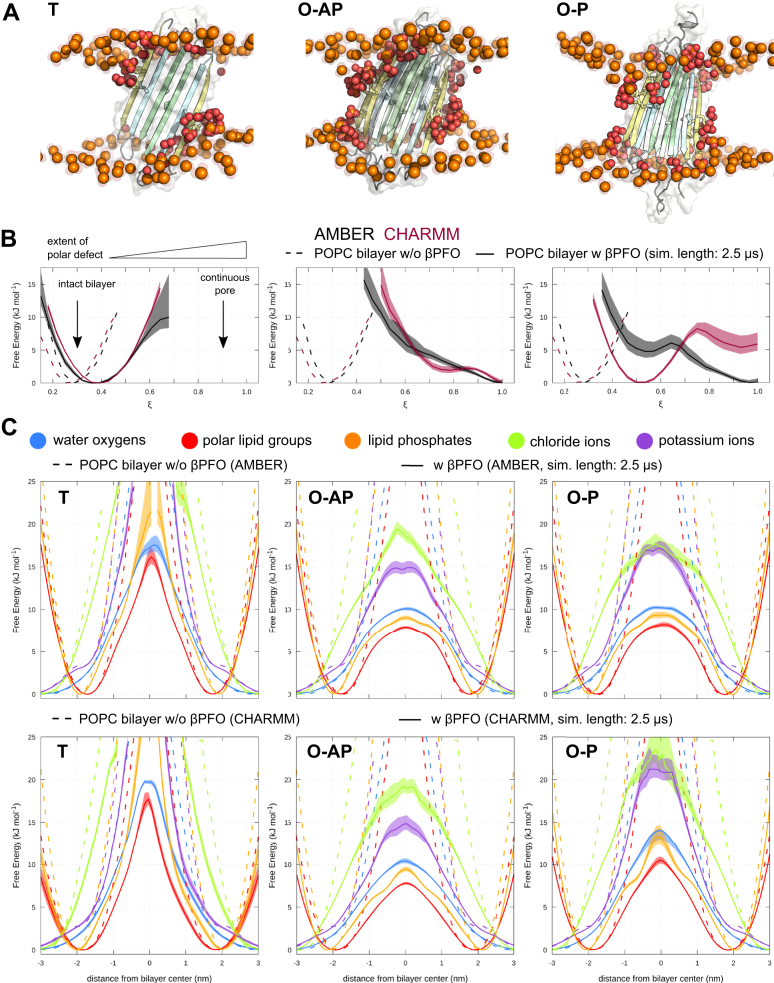
**Extent of A*****β*****42 oligomer–induced membrane permeabilization.***A*, representative snapshots for three A*β*42 oligomer models (T, O-AP, O-P) taken at *t* = 2.5 *μ*s of simulation time with the AMBER force field. Lipid phosphorus (*orange*) and lipid oxygen (*red*) atoms with direct contact to *β*1-strands of the aggregates are shown as colored spheres. The remaining protein and lipid atoms as well as water molecules are not shown for clarity. *B*, distributions of the polar transmembrane defect reported (*ξ*) for AMBER (*black*) and CHARMM (*burgundy*) simulations. Shading indicates the standard error. As reference, the distribution of the continuous polar defect reaction coordinate for an unperturbed POPC bilayer is reported (*broken lines*). *C*, partial density profiles for water (*blue*), polar lipid groups (*red*), lipid phosphates (*orange*), potassium (*purple*), and chloride (*light-green*) ions across the POPC bilayer shown for AMBER and CHARMM simulations. Data are averaged over multiple independent trajectories, shading indicates the standard error. The partial density profiles for an unperturbed lipid bilayer are reported as reference (*broken lines*). POPC, 1-palmitoyl-2-oleoyl-sn-glycero-3-phosphocholine.

The tetrameric and octameric A*β*42 *β*PFO models show a different spontaneous permeabilization behavior consistently in each of the individual simulation runs and among the employed force fields (Figs. 1, *E* and *F*, and 2, *A* and *B*). An increased water permeability of the transmembrane region along either *β*-sheet edge was revealed by simulations of **T** compared to a flat POPC bilayer without embedded A*β*42 *β*PFO (Fig. 2, *A*–*C* and Movie S1). However, no substantial perturbation of the lipid head group region is observed (Fig. 2*C*). The presence of water and lipid oxygen atoms in the hydrophobic center of the membrane was characterized by the polar transmembrane defect in terms of a reaction coordinate analysis (39) (see Experimental procedures, Fig. 2*B*). Accordingly, no polar defects that span the entire lipid bilayer width are seen based on the reaction coordinate *ξ* (Fig. 2*B*). Simulations of the full-length A*β*42 *β*-sheet tetramer and the truncated **T** *β*PFO model show no significant differences in terms of structural stability or water permeability of the bilayer. A slightly higher lipid head group perturbation for the full-length system is observed due to the sporadic and transient formation of partially open pores (Fig. S2, *B* and *C*). Neither the full-length nor the N-terminally truncated A*β*42 pore-forming tetramers induce ion permeation across the POPC bilayer (Fig. S2*A*). To ensure that the observed trends are robust, the **T** *β*PFO simulations with the AMBER force field were extended from 2.5 *μ*s up to 5 *μ*s. Indeed, no eventual pore-opening events occur even on longer time scales (Fig. S3).

In contrast, simulations of both **O-AP** and **O-P** structures show a higher density of water and polar lipid groups in the hydrophobic membrane interior, indicating formation of a hydrophilic pore. The time traces shown in Figure 1*E* illustrate the penetration of water and polar lipid groups in the hydrophobic membrane center, *i.e.* the formation of continuous and stable polar defects within 200 ns for a single representative simulation (see also Movie S2). The **O-AP** *β*-sandwich model most favorably induces fully formed and water filled pores (*ξ* = 1) along its *β*-strand edges (Fig. 2, *A* and *B*) in both tested force fields. The spontaneously formed pores stay open and result in stable transmembrane defects on a multi-*μ*s time scale (Fig. S3). As evident from the partial density profiles in Figure 2*C*, chloride anions and potassium cations also enter into and out of the bilayer center. The position probability distributions show that cations permeate the bilayer more readily during the simulations (Fig. 2*C*). The **O-P** *β*-sandwich model shows a higher permeation barrier for potassium ions (AMBER: by 2.5 kJ/mol, CHARMM: by 7.5 kJ/mol) and an increased fraction of only partially open pores compared to the **O-AP** model (Fig. 2, *B* and *C*).

### The HHQK domain shows specific interactions with lipid head groups

**Figure 3:**
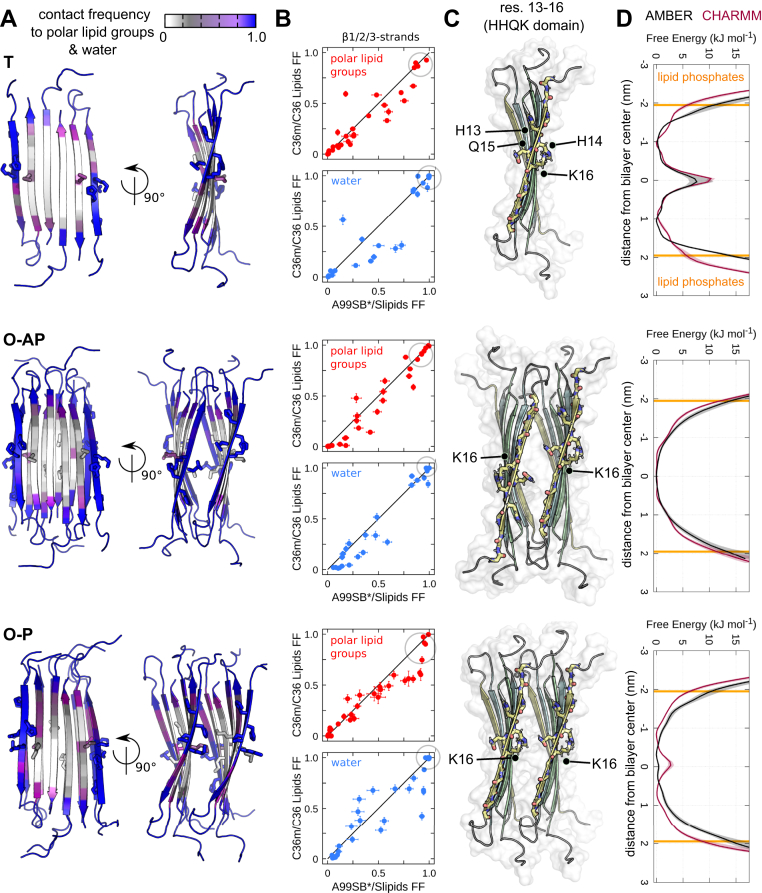
**The N-terminal HHQK domain of A*****β*****42*****β*****PFOs with the 6RHY fold shows specific interactions with lipid head groups.***A*, averaged contact frequencies of polar lipid groups and water molecules to protein residues for three A*β*42 oligomer models (T, O-AP, O-P) mapped onto the initial simulation structure shown in cartoon representation (side and front view). Contact frequencies for each oligomer model are averaged over AMBER and CHARMM simulations, respectively. *B*, detailed breakdown of the average contact frequencies for residues from the transmembrane *β*-strands (*β*1/2/3) to polar lipid groups (*red*) and water (*blue*) are compared for the two tested force fields. The standard error is indicated by error bars. HHQK domain residues are encircled. *C*, renderings of initial simulation coordinates for T, O-AP, and O-P models indicate the position of the HHQK domain (front view). Side-chain atoms for residues 13 to 16 and 35 are shown in *stick* representation. *D*, corresponding density profiles show the location of residues 13 to 16 (HHQK domain) inside the POPC layer. Shading indicates the standard error. βPFO, β-sheet pore-forming oligomer; POPC, 1-palmitoyl-2-oleoyl-sn-glycero-3-phosphocholine.

Residue-based contact mapping shown in Figure 3, *A* and *B* reveals direct interactions between polar lipid head groups and water molecules with the hydrophilic residues 13 to 16 (HHQK domain) of the *β*1-strand, regardless of the initial oligomer model. The simulated **T**, **O-AP,** and **O-P** models slightly tilt during the simulations and show a high average contact frequency of H14 and K16 side-chains to the lipid phosphate groups, whereas the H13 and Q15 side-chains predominantly interact with the positively charged choline group of the lipids. Furthermore, lipid carbonyls show increased contact frequency to the side-chain atoms of Q15 and K16, as well as to the backbone atoms of H14 and K16 due to hydrogen bonding (Figs. S4 and S5). It is of note that the extent of pore formation and pattern of lipid aggregate interactions agree almost quantitatively between both force fields (Figs. 3*B*, S4 and S5). The direct lipid contacts between the HHQK domains on either *β*-sheet edge of the **T** *β*PFO are established on a sub-*μ*s time scale (see Movie S1). The highest density of lipid-contacting HHQK residues is observed approx. 1 nm away from the bilayer center and towards the lipid head group region from opposite membrane leaflets (Fig. 3, *C* and *D*). The layered *β*-sheets in the **O-AP** and **O-P** models feature two HHQK domains on each side of the exposed *β*-strand edges (Fig. 3*C*). The direct interactions of lipid head groups and water with this wider patch of hydrophilic residues facilitate and stabilize the opening of fully hydrated pores (Fig. 3, *C* and *D*).

Continuous polar transmembrane defects are most abundantly induced by the **O-AP** model due to the antiparallel *β*1-strand alignment that results in the HHQK domains spanning the whole center of the bilayer on either edge (Fig. 3, *C* and *D*). The preferential location of the HHQK domains relative to the bilayer center thus rationalizes the varying extent of stable pore formation induced by the tested oligomer models.

Similar to the **T** model, the hydrophobic *β*2- and *β*3-strands of the *β*-sandwich octamer models show no substantial interactions with polar lipid atoms, water molecules, or ions (Figs. S4 and S5). Consequently, the lipid bilayer remains unperturbed along the hydrophobic faces of the *β*-sheet core. The resulting pore shape can be thus best described as a nonconcentric toroid that is restricted to both *β*-sandwich edges (Fig. S6, *A* and *B*). As water or ions are only in contact with the outermost, exposed *β*-sheet edges in the membrane center, no permeation pathway in the aggregate interior is observed in the simulations (Fig. S6*C*).

### A*β*42 *β*PFO stability and pore formation along layered *β*-sheet edges depends on *β*1-strand insertion

**Figure 4:**
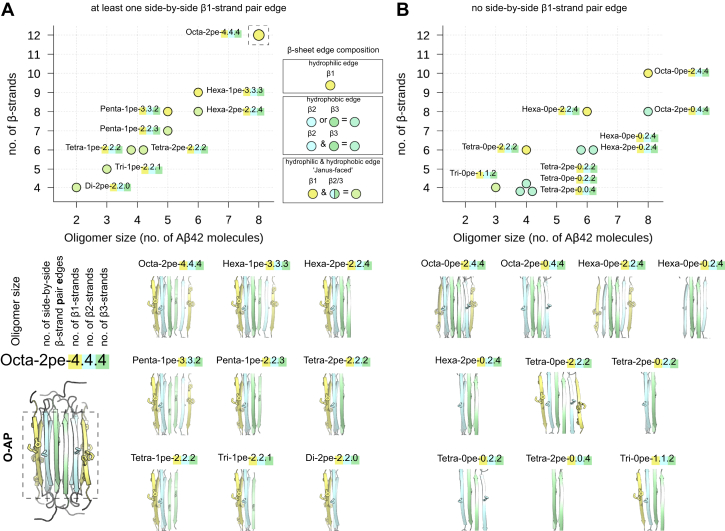
**Summary of A*****β*****42*****β*****PFO models with the 6RHY fold.** Overview over all A*β* oligomers obtained from initial coordinates of the O-AP model. Individual aggregate structures are distinguished by unique identifiers and grouped according to their *β*-sheet edge conformation: (*A*) A*β*42 models with at least one side-by-side *β*1-strand pair edge and (*B*) A*β*42 models without side-by-side *β*1-strand pair edge. For each system, the oligomer size (number of A*β*42 molecules) and the number of *β*-strands is plotted. *Circle* colors were chosen to match the composition of the outermost *β*-strands, *i.e.* the *β*-sheet edges of the 6RHY oligomer models: all *β*1 (hydrophilic) - *yellow*, all *β*2/*β*3 (hydrophobic) - *cyan*, *β*1 and *β*2/*β*3 (’Janus-faced’) - *green*. βPFO, β-sheet pore-forming oligomer.

**Figure 5:**
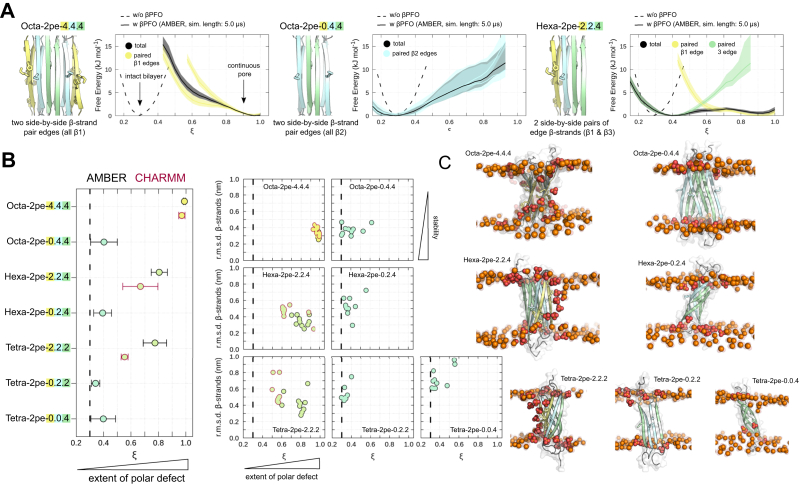
**A*****β*****42*****β*****PFO stability and pore formation along layered *β*-sheet edges depends on N-terminal insertion.***A*, starting structures of up-down, face-to-face (*left*) octameric and hexameric *β*-sandwich oligomer models (*center*) with and (*right*) without *β*1-strand edges. Accompanying panels report the occurrence of polar transmembrane defects (*ξ*) averaged over multiple independent simulations. Distributions are reported in total (*black line*) and separately along each individual *β*-strand edge (*colored lines*). *B*, the average polar transmembrane defect is shown for a total of seven *β*-sandwich oligomer models with layered *β*-sheet edges, with and without side-by-side *β*1-strand pair edges (left panel). Symbols indicate averages over all independent simulations per oligomer model, error bars denote the standard error. The symbol outline colors indicate the force field used (*black* - AMBER; *burgundy* - CHARMM). Additional symbol annotations are the same as in Figure 4. The average rmsd of the transmembrane *β*-strands with respect to the simulation starting structure are shown as function of the average polar transmembrane defect (right panels). The symbols report individual averages for each independent simulation per oligomer model. *C*, representative snapshots of selected simulations illustrate the extent of the polar defect and conformational deviation for each oligomer model shown in (*B*). βPFO, β-sheet pore-forming oligomer.

The ability of A*β*42 octamers with layered *β*-sheet edges to induce pore formation is associated with characteristic lipid contacts *via* residues 13 to 16 (HHQK) from the N-terminal *β*1-strand. This led us to further probe the stability of A*β*42 *β*PFO models and their capacity for pore formation with a particular focus on the role of the peptide’s N-terminus. To do so, aggregate isoforms were derived by selectively removing the HHQK domain bearing *β*1-strands from the TMD of the octameric *β*-sandwich model structure to mimic a 6RHY type aggregate without membrane-inserted N-terminus (Figs. 4 and 5*A*). We obtained an octameric *β*-sandwich model with layered *β*-sheet edges formed only by the hydrophobic *β*2/*β*3 strands from the C-terminus of A*β*42 (Figs. 4 and 5*A*). Based on the previous analysis, only the **O-AP** model was considered a good template for the smaller A*β*42 *β*PFOs with the 6RHY fold (Fig. 4). We also tested tetrameric and hexameric *β*-sandwich models with and without N-terminal truncation (Fig. 4*A*). The latter, ’Janus-faced’ *β*-sandwich aggregates, exhibit by design a hydrophilic side-by-side *β*1-strand pair edge on one side (identical to the octamer) and a hydrophobic side-by-side *β*2/3-strand pair edge on the other side of its TMD structure (Figs. 4 and 5*A*). As expected from the previous findings, also the smaller, ’Janus-faced’ *β*-sandwich conformations form continuous polar transmembrane defects along the side-by-side *β*1-strand pair edge with a high propensity. Yet, independent of tested oligomer size and conformation, no pore formation occurs along side-by-side pair edges formed by *β*2/*β*3-strands (Fig. 4*A*).

The stability of the TMD *β*-strands in terms of rmsd and capacity to form continuous polar membrane defects of the tested aggregate model structures is summarized in Figure 5*B*. A*β*42 *β*-sandwich oligomers without any intra-membrane, N-terminal *β*1-strands do not form pores and cause only small, if any detectable perturbations of the lipid bilayer in the simulations (Fig. 5*B*). The *β*-sandwich octamer with *β*1-strands is the most stable among the tested oligomer models, maintaining the high initial *β*-sheet secondary structure content throughout the simulated *μ*s time scale. All smaller oligomers show sizable deviations from the initial structural models (Fig. 5*C*). The ’Janus-faced’ tetrameric and hexameric *β*-sandwich conformations are less stable than the octamer model in AMBER and CHARMM simulations, due to frequent separations of the mated *β*-sheets at the *β*2/*β*3-strand edge. The *β*PFO derivatives with layered *β*-sheets composed only of hydrophobic *β*2/*β*3-strands do not appear to be stable either (Fig. 5, *B* and *C*). Instead, even the larger *β*PFO models with six or eight TMD *β*-strands show twisted and sheared *β*-sheet layers. We also observed the conversion to single *β*-sheet and *β*-barrel–like states, in each case resulting in an increased amount of interchain *β*-sheet contacts (Fig. 5*C*).

### A*β*42 oligomers without layered *β*-sheet edges are not able to form stable pores

**Figure 6:**
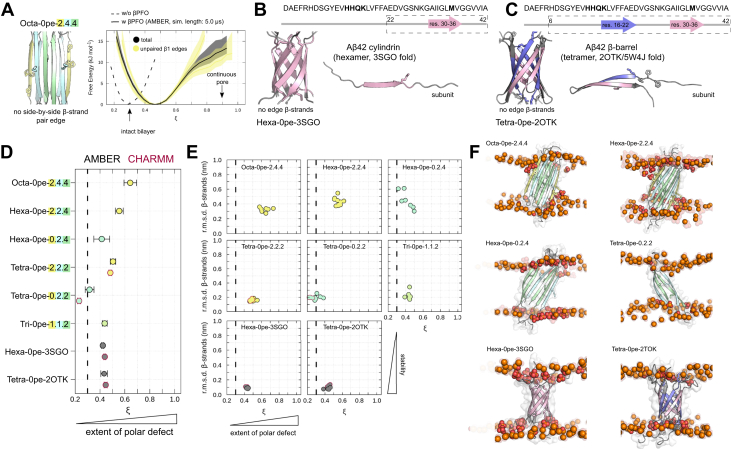
**A*****β*****42 oligomers without layered *β*-sheet edges do not form stable pores.***A*, starting structure of up-down, face-to-face octameric *β*-sandwich oligomer model without layered *β*-sheet edges. Accompanying panels report the occurrence of polar transmembrane defects (*ξ*) averaged over multiple independent simulations. Distributions are reported in total (*black line*) and separately along each individual *β*-strand edge (*colored lines*). Models of a (*B*) hexameric and (*C*) tetrameric A*β*42 oligomers derived from *β*-barrel structure templates without any *β*-sheet edges. *D*, the average polar transmembrane defect is shown for a total of 8 A*β*42 oligomer models without layered *β*-sheet edges or without any *β*-sheet edges (*left panel*). Additional symbol annotations are the same as in Figure 4. *E*, the average rmsd of the transmembrane *β*-strands with respect to the simulation starting structure is shown as a function of the average polar transmembrane defect (*right panels*). *F*, representative snapshots of selected simulations illustrate the extent of the polar defect and conformational deviation for each oligomer model.

To examine whether side-by-side *β*1-strand pair edges are necessary to induce spontaneous pore formation, we studied additional A*β*42 *β*PFO models without layered *β*-sheet edges, varying in number and composition of their TMD *β*-strands (Fig. 4*B*). The respective *β*-sheet aggregates were obtained by selectively removing *β*1/*β*2-strands from the **T** and **O-AP** 6RHY structure models (Fig. 4*B*). Deleting a single *β*1-strand on either edge of the **O-AP** is sufficient to significantly diminish the otherwise high membrane permeability induced by this oligomer (Fig. 6*A*). The extent of transmembrane polar defects decrease linearly upon removal of further *β*-strands from octameric to trimeric structures (Fig. 6*D*). In fact, none of the tested oligomeric states without layered *β*-sheet edges are able to form continuous pores or large polar defects in the membrane.

Independent of aggregate size, the majority of structure models is stable on the multi-*μ*s simulation time scale as judged by the small rmsd of the TMD *β*-strands (Fig. 6*E*). The **T** model, which is also the building block of the larger tested asymmetric *β*-sandwich oligomers, is the most stable. The hexameric, asymmetric *β*-sandwich oligomer without membrane-inserted *β*1-strands (Hexa-0pe-0.2.4) is the least stable aggregate model and equilibrates towards single *β*-sheet and *β*-barrel-like states, similar to what is observed for the symmetric *β*-sandwich structures entirely composed of the hydrophobic, C-terminal TMD *β*-strands.

Our results indicate that (*β*-sandwich) aggregate conformations are unstable in the absence of polar lipid contacts to the entire length of the intramembrane *β*-sheet edges. Instead, closed arrangements are favored, where the edges of both *β*-sheet layers establish hydrogen bonds with one another. To further test this premise, we sought to examine preformed *β*-barrel oligomers that are not based on the 6HRY structure model. We selected a tetrameric *β*-barrel (Tetra-0pe-2OTK derived from PDB ID: 2OTK/5W4J) and hexameric cylindrin (Hexa-0pe-3SGO derived from PDB ID: 3SGO) with different folds as additional structure models for A*β*42 *β*PFOs without any *β*-sheet edges (see Experimental procedures, Figs. 6, *B* and *C* and S7). The tetrameric *β*-barrel based on 2OTK A*β* hairpin conformations as principal structural motif shows a high degree of stability during the simulations. The cylindrin-like hexamer structure formed by the C-terminal part of the A*β*-peptide (37) is also very stable within POPC bilayers. However, both models show no pore formation or capacity to permeate ions across the lipid bilayer. The *β*-barrel structures are devoid of water contacts in the center of the membrane, indicating that also no aqueous interior pores are present (Figs. 6*C* and S8 and S9).

### Phospholipid interactions stabilize A*β*42 oligomer models with the 6RHY fold

**Figure 7:**
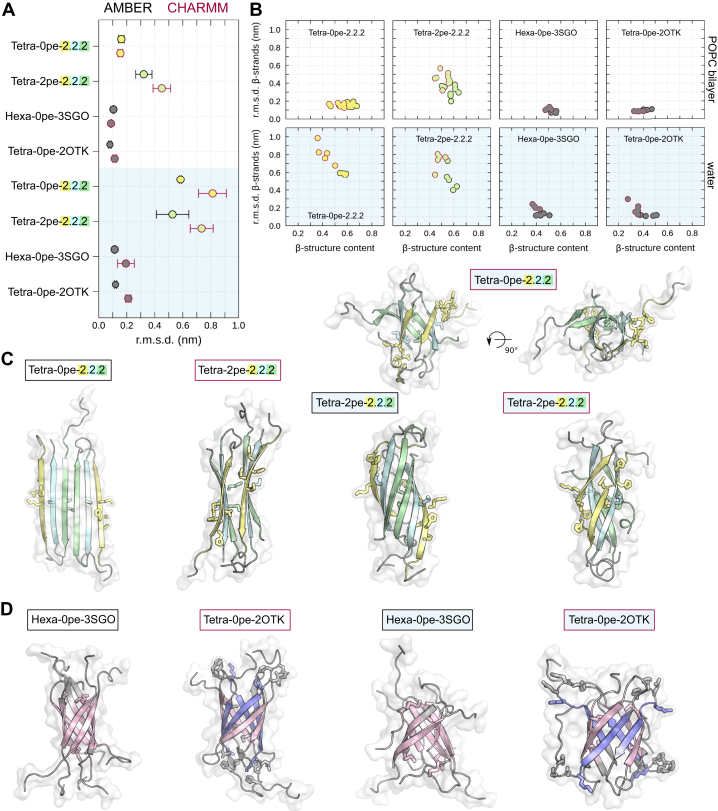
**The phospholipid environment stabilizes A*β*42 oligomer models with the 6RHY fold.***A*, the panel reports the average rmsd of the transmembrane *β*-strands with respect to the simulation starting structure for tetrameric *β*-sheet and *β*-sandwich models, as well as hexameric and tetrameric A*β*42 *β*-barrel models simulated with and without a phospholipid environment. Additional symbol annotations are the same as in Figure 4. *B*, the average rmsd of the transmembrane *β*-strands are shown as a function of the average *β*-structure content for all independent trajectories. Simulation data of A*β*42 oligomer models performed in water are highlighted by shaded background. Snapshots of selected simulations with (*C*) 6RHY fold and (*D*) *β*-barrel structures without any *β*-sheet edges illustrate the effect of the simulation environment (immersed in POPC bilayer vs water only) on the structural stability. POPC, 1-palmitoyl-2-oleoyl-sn-glycero-3-phosphocholine.

A comparison of tetrameric oligomer conformations simulated in aqueous solvent and POPC lipid bilayers indicates that lipid–protein interactions have a significant stabilizing effect on *β*-sheet and *β*-sandwich tetramers with the 6RHY fold (Fig. 7*A*). In particular, the **T** model, while stably immersed in a phospholipid bilayer, converts to more compact and curved aggregates in the absence of lipids and shows a partial loss of extended *β*-structure content (Fig. 7, *B* and *C*). The Tetra-0pe-2.2.2 and Tetra-2pe-2.2.2 models form incomplete *β*-barrel topologies with increased hydrogen bonding at the *β*-sheet edges (Fig. 7, *B* and *C*). Interestingly, the tetrameric and hexameric A*β*42 *β*-barrel models (Fig. S7) without free edge *β*-strands display a stability in water comparable to the membrane-embedded structures (Fig. 7, *B* and *D*). Both AMBER and CHARMM force fields show the same trends in terms of solvent media (lipid bilayer or water)–dependent aggregate stability for the tested structure models (*β*-sheet, *β*-sandwich, or *β*-barrel) with evidence of a higher overall stability in the AMBER simulations (Fig. 7*B*).

### Pore formation and ion permeation occurs only along stable *β*-sheet pair edges but independent of oligomer size

**Figure 8:**
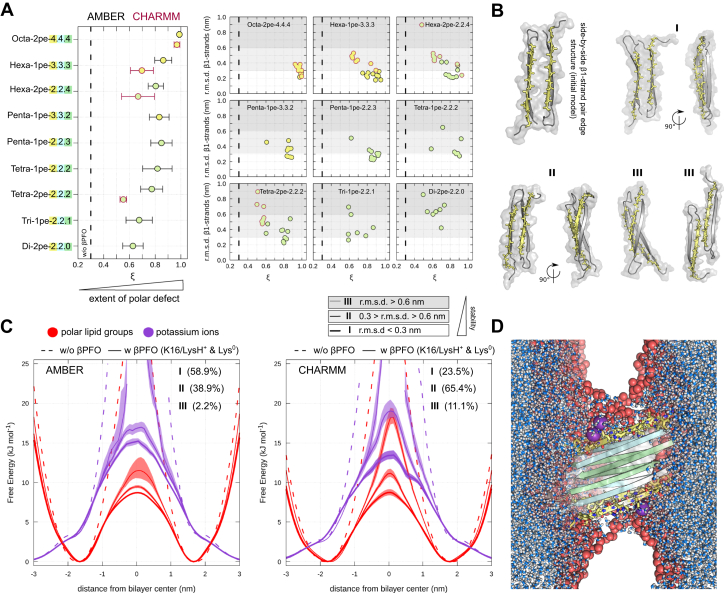
**Formation of ion-conducting pores depends on side-by-side *β*1-strand pair edge stability.***A*, the average polar transmembrane defect is shown for a total of 9 A*β*42 oligomer models with at least one side-by-side *β*1-strand pair edge (*left panel*). Additional symbol annotations are the same as in Figure 4. The average rmsd of the side-by-side *β*1-strand pair edge with respect to the simulation starting structure (depicted in *B*) are shown as function of the average polar transmembrane defect (*right panels*). *B*, front and side views of selected simulation snapshots illustrate the deviation from the initial side-by-side *β*1-strand pair edge conformation taken from individual A*β*42 *β*PFO models (rest of aggregate not shown). *C*, average partial density profiles for polar lipid groups and potassium ions are shown as a function of side-by-side *β*1-strand pair edge stability. Conformations from AMBER (left) and CHARMM (right) simulations are grouped based on rmsd in three pools (I, II, III). Shading indicates the standard error. Relative conformation populations within the three pools are provided. *D*, representative O-AP simulation snapshot shows multiple potassium ions (*purple spheres*) spontaneously entering the lipid bilayer center. Lipid oxygen atoms are shown as *red spheres*. βPFO, β-sheet pore-forming oligomer.

To corroborate the emerging trends, we conducted a systematic investigation of smaller *β*PFOs (dimers to hexamers) with at least one side-by-side *β*1-strand pair edge (Fig. 4*A*). As done before, subsets of the TMD *β*-strands from the **O-AP** template were selected to arrive at the respective 6RHY *β*PFO model structures. Together with the three oligomer models presented in Figure 5*A*, a total of nine A*β*42 *β*PFO models with at least one side-by-side *β*1-strand pair edge were compiled (Fig. 4*A*). The stability of the *β*PFO aggregate models, specifically of the side-by-side *β*1-strand pair edge, shows a striking correlation with the ability to form stable, aqueous pores (Fig. 8*A*). Aggregates larger than the trimer form stable hydrated edge pores during the majority of all individual multi-*μ*s long simulations. However, also smaller dimeric and trimeric *β*PFO aggregates retain stable side-by-side *β*1-strand pair edges in multiple simulation runs, resulting in pore formation (Fig. 8, *A*). Interestingly, the lateral addition of a *β*1/*β*2-strand hairpin to the tetrameric *β*-sheet model (**T**) is already sufficient to create a stable, pore-forming side-by-side *β*1-strand pair edge motif (Figs. 4*A* and 8*A*, Penta-1pe-3.3.2).

The simulations reported in Figure 8*A* were carried out with charged lysine side-chains at position 16 compatible with pH 7.4 (K16/LysH^+^). We note that the **O-AP**, Hexa-1pe-3.3.3, and Tetra-2pe-2.2.2 models with two neutral K16/LysH^0^ (pH 9.0) also show membrane perturbing characteristics. However, the extent of pore formation and HHQK domain interactions with the lipid head group region is decreased compared to simulations with ionized K16 (Fig. S10). The **O-AP** and Hexa-1pe-3.3.3 aggregate models with neutral K16 side-chains retain conformations that are similar to the ones with ionized K16 or stay even closer to the initial structure model in both force fields (Fig. S11).

Figure 8*C* shows the correlation between the rmsd of the side-by-side *β*1-strand pair edge and extent of edge conductivity considering K16/LysH^+^ and K16/LysH^0^ simulations. The most stable layered *β*-sheet edges show the lowest energetic barrier for potassium ions entering the hydrophobic bilayer center (Fig. 8, *C* and *D*). In contrast, no ion permeation is registered when the side-by-side *β*1-strand pair edge conformations exhibit a rmsd higher than 0.6 nm from the initial structure (Fig. 8, *B* and *D*). In the simulations, such high rmsd values are caused, *e.g.*, by twisting and shearing of the initially parallel *β*-sheet layers that separate both HHQK domains. Once the side-by-side *β*1-strand structure deforms further or closes due to interstrand hydrogen bond formation, the edge conductivity is lost and not compatible with ion permeation anymore (Fig. 8, *B* and *C*). The pore-forming characteristics of well aligned, layered *β*-sheet edges with persistent close intersheet contacts between the two HHQK domains (low rmsd) are consistent in AMBER and CHARMM simulations. The underlying distributions of *β*PFO model conformations are, however, skewed differently (Fig. 8*C*).

For smaller *β*PFO models, the difference in the stability of the side-by-side *β*1-strand pair edge is more pronounced in the CHARMM force field and sheds light on the force field dependence of the simulation outcome (Figs. 8*A* and S12). In particular, the tetrameric *β*-sandwich conformations (Tetra-2pe-2.2.2) are less stable in CHARMM simulations than the AMBER simulations (Fig. S12, *A*–*C*). Indeed, pore-forming structures sampled during AMBER simulations and spawned in the CHARMM force field reequilibrate within 2 *μ*s towards conformations that do not perturb the bilayer significantly (Fig. S12, *D* and *E*).

In conclusion, our simulations indicate that aggregate stability is likely governed by nuanced lipid–protein and protein–protein interactions and may be force field dependent for smaller, metastable *β*-sandwich oligomer models.

### Ion conducting properties of lipid-stabilized pores is defined by specific interactions with side-by-side *β*1-strand pair edge

**Figure 9:**
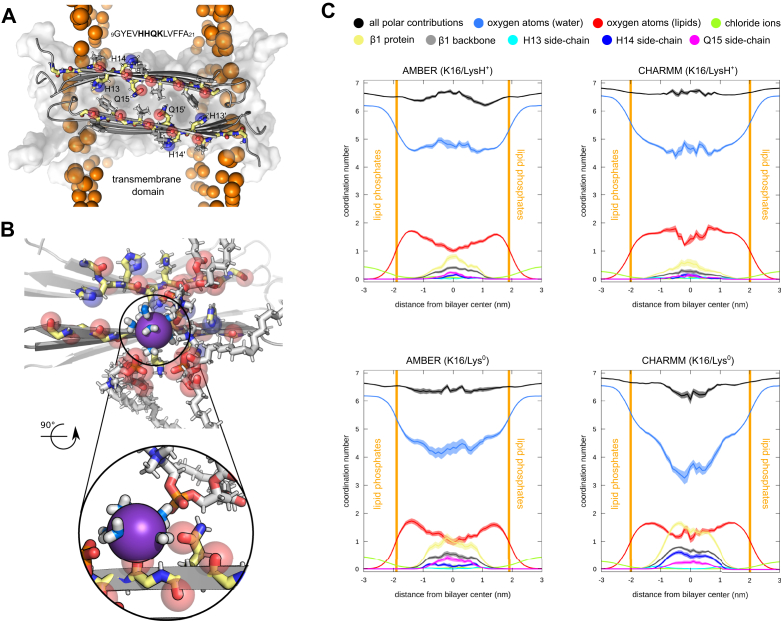
**Ion permeability is facilitated by direct and specific interactions with side-by-side *β*1-strand pair edge.***A*, the side-by-side *β*1-strand pair edge structure is shown with all backbone atoms, additionally side-chain atoms of residues 12 to 20 are depicted by *sticks*. Carbonyl oxygen atoms from the backbone and from polar side-chains are highlighted by *transparent spheres*. Lipid phosphorus atoms are shown as *orange spheres*. *B*, simulation snapshot of the side-by-side *β*1-strand pair edge structure in contact with a potassium ion entering the hydrophobic region around the membrane center. A close up view of the ion’s direct atomic contacts to protein, lipid, and water molecules are shown. *C*, the average composition of the first hydration shell of potassium ions spontaneously permeating across the POPC bilayer is shown as a function of the K16 protonation state in two force fields (*left*, AMBER and *right*, CHARMM). The average coordination number of the cation is plotted as a function of the ion position along the bilayer axis. Shading indicates the standard error. The sum of all direct contacts with protein atoms (*yellow*) are shown together with individual coordination numbers: backbone carbonyls of residues 9 to 21 (*gray*), as well as H13 (*cyan*), H14 (*dark-blue*), and Q15 (*magenta*) side-chains. POPC, 1-palmitoyl-2-oleoyl-sn-glycero-3-phosphocholine.

To disentangle individual contributions to cation permeation in the membrane center, we monitored direct protein contacts to potassium ions crossing the bilayer in tetrameric, hexameric, and octameric *β*PFO models, all of whom feature at least one antiparallel side-by-side *β*1-strand pair edge structure. Figure 9*A* shows the side-by-side *β*1-strand pair edge up close and highlights the carbonyl oxygens, along with the histidine (H13, H14), glutamine (Q15), and phenylalanine (F19, F20) side-chains.

Backbone and side-chain atoms from residues 9 to 21 comprising the *β*1-strand exhibit transient but direct contacts to potassium ions as found from a contact analysis (Fig. S5). Residues H13 and F20, located at opposite ends of the *β*1-strand and close to the lipid-water interface, have the highest average contact frequency (Fig. S5). Residues H14, Q15, and F19 show direct cation contacts, as well. An example of a potassium permeation event in the bilayer center with direct backbone and side-chain atom interactions is depicted in Figure 9*B* and Movie S3, showing that side-chain and backbone carbonyls can also coordinate the monovalent cation in concert. We calculated the average composition of the first hydration shell of potassium ions with a distance cutoff of 0.35 nm. A hydration profile of potassium ions crossing the bilayer along the side-by-side *β*1-strand pair edges for AMBER and CHARMM simulations is shown in Figure 9*C*. Hydrated potassium ions (bulk) maintain a high number of surrounding waters upon entering the transmembrane region due to the polar nature of the formed pore. The ions experience a loss of one to two coordinating waters in the membrane center on average. The lost water coordination is compensated by contacts with polar lipid and protein atoms. Notably, in the center most part of the bilayer, exposed carbonyl oxygens from the protein backbone (*β*1-strand) and Q15 side-chain atoms contribute directly to the coordination sphere of permeating potassium ions (Fig. 9*C*). Similarly, close contacts between the potassium ions and the imidazole nitrogen from the H13 and H14 side-chain were observed.

The K16/LysH^0^ simulations show that the K16 protonation state does influence the ion coordination patterns, showing increased contacts to the *β*1-strand for permeating ions (Fig. 9*C*). Concordantly, fewer water and lipid oxygens coordinate the potassium ions moving across the bilayer center than K16/LysH^+^, in particular for CHARMM simulations.

### Mutations in the *β*1-strand emphasize the specificity of *β*-sheet edges in A*β*42 *β*PFOs

In subsequent computer experiments, we aimed to explore the specificity of the layered *β*-sheet edges formed by A*β*′s N-terminus. Specifically, we tested how single residues impact the pore forming and ion conduction ability of these *β*PFO models. Experimental data suggest that certain mutations in the *β*1-strand likely affect the cytotoxicity compared to the WT A*β* peptide. The F19G mutation, for instance, was shown to abolish toxicity (40, 41). An attenuation of toxicity was also observed by altering amino acids 13 through 17 (GGQGL) (42) or through methylation of nitrogen atoms from the imidazole rings of H13 and H14 (43). In contrast, a 1:1 mixture of K16N and WT A*β* produces highly toxic oligomers (44).

**Figure 10:**
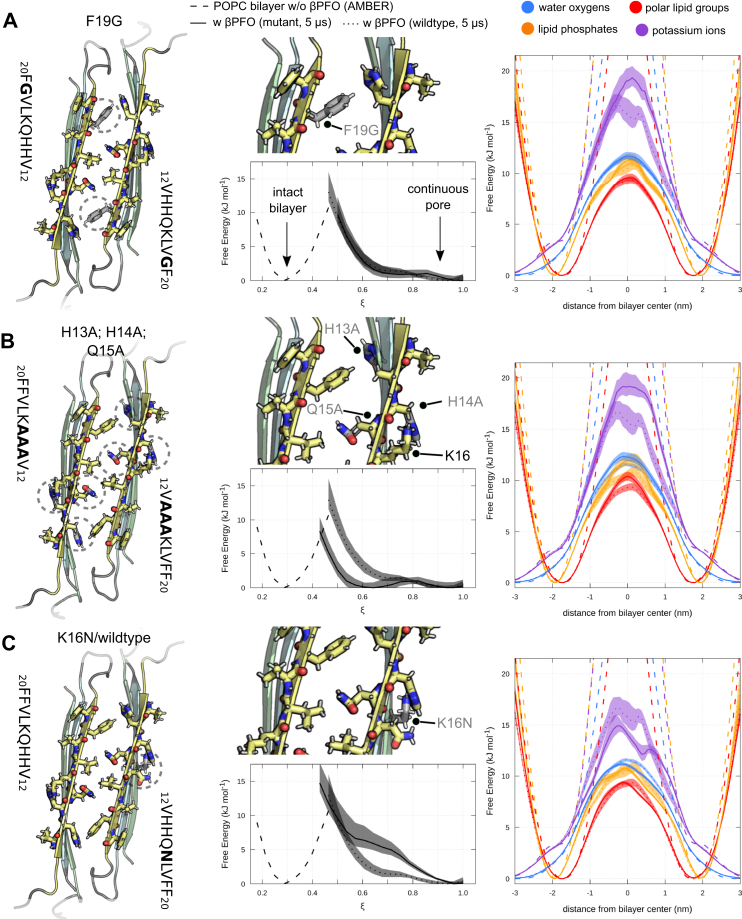
**Point mutants emphasize the specificity of the side-by-side*****β*****1-strand pair edge structure and HHQK domain.** Front view of side-by-side *β*1-strand pair edge structure for (*A*) F19G, (*B*) H13A; H14A; Q15A, and (*C*) K16N/WT mutants of the hexameric *β*-sandwich oligomer (Hexa-2pe-2.2.4). Locations of mutated residues are indicated by *dashed circles* and shown in close-up (*center panels*). Side-chain atoms of the WT oligomer model are shown as *gray sticks* in the background for comparison. Extent of transmembrane defects and partial density profiles of water (*blue*), all polar lipid groups (*red*), lipid phosphates (*orange*), and potassium ions (*purple*) across the POPC bilayer are shown for each mutant, respectively. As reference, the partial density profiles and polar defect reaction coordinate for an unperturbed POPC bilayer (*broken lines*) and the WT hexameric *β*-sandwich oligomer model (*dotted lines*) are also reported. Shading indicates the standard error. POPC, 1-palmitoyl-2-oleoyl-sn-glycero-3-phosphocholine.

We simulated these three sets of mutations for the hexameric *β*-sandwich model (Hexa-2pe-2.2.4): The F19 side-chain (40, 41) is located on opposite ends of the side-by-side *β*1-strand pair edge (Fig. 10*A*), whereas the H13A; H14A; Q15A (Fig. 10*B*) and K16N (44) (Fig. 10*C*) mutations directly affect the HHQK domains and either delete or introduce a side-chain carbonyl group. All probed variants show a minor impact on the extent of pore formation and stability of the 6RHY fold but change the ability to permeate ions across membrane compared to the WT A*β*42 *β*PFO model. The F19G and alanine mutations caused a significant reduction in ion permeation, increasing the energetic barrier by more than 4 kJ/mol, respectively (Fig. 10, *A* and *B*). The K16N mutant increase the capacity of the hydrophilic transmembrane *β*-sheet edge to bind ions (Fig. 10*C*), thus lowering the energetic barrier in the bilayer center. This observation agrees with the previously discussed direct residue-ion contacts, in particular with the cation coordination of the side-chain carbonyls (Fig. 9*C*). The impact of deleting the inward facing aromatic residue phenylalanine (F19) and both histidines (H13/H14) in the side-by-side *β*1-strand pair edge furthermore points to a relevance of cation-pi interactions for the entry of ions in the transmembrane region.

## Discussion

Small, oligomeric, and membrane-bound structures may represent the most neurotoxic aggregates during A*β* misfolding and self-assembly (2, 6, 8, 9, 11, 13, 16, 19, 20, 21, 26, 28). Determining the mechanistic underpinnings of pathological membrane permeabilization from a structure-activity standpoint, however, remains a considerable challenge due to the scarcity of experimentally determined atomic structures. MD simulations allow to investigate isolated oligomeric states and gauge their conformational stability and dynamical interactions based on 3D structure models. Several theoretical models of A*β* oligomers have been presented in the past: de novo assembled from A*β* monomers (45, 46, 47, 48), by threading the A*β* sequence on *β*-barrel and cylindrin-like scaffolds (37, 49), or by modeling of A*β* conformations based on fibrillar cross-*β* structure motifs (50, 51). An in-depth review of computer simulations on A*β* oligomer models in solution or bound to model membranes is given elsewhere (48, 52).

Here, we probed the principal relationship between membrane permeabilization and 3D structures of low molecular-weight A*β*42 oligomers, ranging from dimer to octamers in size, using all-atom MD simulations. Only membrane-embedded A*β*42 oligomers with the 6RHY fold and exposed edges of mated *β*-strands from the A*β* N-terminus spontaneously form and maintain continuous, lipid-stabilized pores. By comparing the behavior of A*β*42 truncation variants and isoforms, we demonstrate that *β*-sandwich models with the 6RHY fold lacking a membrane-inserted N-terminus do not form pores and show a significantly reduced stability. We suggest a specific mechanism of ion permeation across edge-conductivity pores involving direct protein-ion contacts with side-by-side pair of *β*1-strands, as well as an interdependence between the overall aggregate stability and the anchoring of the HHQK domain in the membrane.

Among the tested oligomer 6RHY models, the tetrameric *β*-sheet oligomer and octameric *β*-sandwich with identical subunits are the most stable, in line with the experimentally observed homogeneous A*β*42 oligomer size populations (29, 33). Based on our simulation results, the tetramer *β*-sheet model (**T**) with the 6RHY fold could play a crucial role in the formation of larger A*β*42 *β*PFOs. The tetramer *β*-sheet was stable and invariably anchored in the two-dimensional matrix of phospholipid bilayers. This could effectively reduce the combinations of available aggregate packings in the nucleation stage and thus provide the necessary templates to facilitate the octameric *β*-sandwich structure formation. We additionally found that the tetrameric *β*-sheet could serve as stable building block in pentameric and hexameric oligomers that form by lateral addition of individual A*β* molecules. The simulations furthermore support the notion of at least metastable subpopulations of dimers, trimers, and tetramers with a side-by-side pair edge motif of *β*1-strands. It is known that phospholipids are crucial for the formation and stability of A*β*42 oligomers (17, 29, 30, 33). The presence of lipid membranes and their composition modulates A*β* aggregate structures and toxicity (19, 29, 30, 33, 53). Our study provides additional evidence that tetrameric *β*-sheet and *β*-sandwich conformations with the 6RHY fold are only stable as lipid–protein complexes and significantly less stable in aqueous solution. Our findings therefore suggest that edge pore formation may entail favorable lipid–protein interactions, critical in stabilizing the distinct features of A*β*42 oligomers with the 6RHY fold. Moreover, tight and specific lipid–protein interactions as found here would also be consistent with detergent-like effects of A*β* oligomers (17, 21). The hydrophobic C-terminal part of A*β* is known to be important for the intramembrane self-assembly pathway (18) and is found as the key constituent of several A*β*42 oligomer structures with intermolecular antiparallel *β*-sheets (29, 36, 54). All tested A*β*42 aggregate models without the membrane-interacting *β*1-strand exhibit a significant amount of *β*-structure, however, are restricted to conformational states with compact *β*-barrel–like and twisted *β*-sheets. The structural conversion of truncated 6RHY oligomers without *β*1-strands highlights interstrand hydrogen bond formation as a dominant driving force for transmembrane *β*-strand conformations in the absence of additional lipid–protein interactions (55). Osterlund *et al*. reported that incubation of A*β* monomers in a micellar environment results in A*β*42 oligomer sizes up to hexamers, whose collisional cross sections determined by native mass spectrometry data are compatible with isotropic or *β*-barrel structures (30). The underlying building block chosen for the putative structural model was a 2OTK *β*-turn-*β* fold (56) that does not involve the peptide’s N-terminal part in the *β*-sheet core (30). From additional simulations of a tetrameric *β*-barrel based on 2OTK A*β* hairpin conformations and preformed cylindrin-like hexamers (37), we conclude that closed, antiparallel *β*-sheet arrangements of membrane-inserted A*β* molecules represent a conformational state with high relative stability, both in lipid bilayers and aqueous solvent. Despite the comparable stability of these oligomer states, a considerable degree of variability, *e.g.* in the number of *β*-strands and *β*-sheet topology may explain the structural polymorphism observed in low molecular-weight A*β* oligomers (3, 11, 29, 30, 33, 34, 36, 37, 54).

The current study provides evidence that small A*β* oligomers show a common membrane permeabilization mechanism by formation of edge conductivity pores involving exposed *β*-strand layers from the N-terminus. Stable edge pore formation was observed for a number of oligomer sizes, ranging from tetramers to octamers, however, only if stable, exposed *β*-sheet edge double layers are present. The continuous lining of hydrophilic residues 13 to 16 (HHQK domain) found in the antiparallel pairings of the *β*1-strand edge provides an intuitive explanation for the observed difference in pore formation ability for tetramer *β*-sheet and octamer *β*-sandwich models. Previous studies have implicated the HHQK domain in the formation of stable intramembrane A*β* oligomers (18). Mutations in this region of the A*β*42 sequence that disrupt protein–lipid interactions were shown to abolish pore formation (42, 57).

We have investigated the permeation mechanism of monovalent potassium ions for edge pore-forming A*β*42 oligomers with the 6RHY fold and found direct protein–ion interactions *via* carbonyl groups from the exposed protein backbone and side-chain atoms from the HHQK domain. The octameric *β*-sandwich models with two double layer *β*-sheet edges represent the most conductive states in terms of ion permeation. Our findings indicate that the ability to induce stable, toroidal shaped transmembrane defects is a necessary prerequisite for ion permeation. Membrane permeabilization of ionic species, however, is sensitive to distortions of layered *β*-sheet edge conformation, as well as to mutations in the N-terminal *β*1-strand. We simulated several mutations that are known to either impair (F19G and H13A, H14A, Q15A) or enhance (K16N) A*β* toxicity (41, 42, 44). Interestingly, these mutations decrease or increase the number of favorable side-chain ion contacts in the membrane-inserted N-terminal *β*1-strand as observed in the simulations and affect the extent of ion permeation into the bilayer center accordingly. Previous *in vitro* studies demonstrated the inhibition of A*β*42 oligomer-induced ion conductance and toxicity by binding of small peptide fragments to the HHQK domain (14) and treatment with histidine-associating compounds (58). We conclude that direct and specific protein interactions exist along the ion-conducting pathway. The permeation process is therefore governed by A*β*42 oligomer edge interactions beyond the attraction of hydrophilic lipid head groups into the membrane center that stabilize the pore. It is of further note that the N-terminal part of A*β* is also well known to bind divalent metal ions, such as Zn^2+^ and Cu^2+^ (59, 60), which are found to enrich in A*β* plaques.

Specifically, H13 and H14 from two adjacent A*β* monomers were shown to coordinate with Zn^2+^ ions (59), rapidly inducing toxic, off-pathway A*β*42 oligomers (61). The side-by-side pair edge of *β*1-strands in both tested packing modes (parallel or antiparallel *β*1-strand alignment) results in two HHQK domains in close proximity, compatible with such a scenario. Therefore, it would be interesting to investigate the conformation-specific interactions of divalent metal ions to 6RHY type oligomers in greater detail.

We would like to stress that none of the tested low molecular-weight A*β*42 oligomer models, varying in size and *β*-sheet topology, exhibited a membrane permeabilization mechanism with an interior pathway for either water or ions. A*β*42 *β*PFO models may differ in structure and permeation mechanism depending on the respective oligomer size distribution. Previously suggested pore models (17, 36, 50, 62, 63) do not feature exposed transmembrane *β*-sheet edges and often lack the N-terminal domain altogether (30, 36, 50). Ion channel–like A*β*42 structures with inner pores that are sufficiently stable and wide to conduct solvated ions would likely require folding into highly regular and long transmembrane *β*-barrels (62, 63) or formation of much larger, high molecular-weight assemblies with annular or concentric pores (50, 63). Atomistic MD simulations of a number of putative membrane-embedded A*β* structures (hepta-, octa-, and 16-mer *β*-barrels) furthermore show that oligomers formed by the C-terminal part of A*β* and with mainly hydrophobic inner surface area fail to support stable aqueous pore formation in lipid membranes (48). The present simulations corroborate and refine on A*β*42 oligomer–phospholipid interactions and induced water permeation across the transmembrane region findings from MD simulations using CHARMM36 force field parameters only with order-of-magnitude longer simulations on the *μ*s time scale (33). Compared to the previous simulations of 6RHY oligomer models (33), we did not apply a transmembrane voltage in the current study to circumvent pore initiation or pore expansion by electroporation effects.

Our results on pore formation and ion permeation (*e.g.* partial densities, lipid-protein contacts, ion coordination profiles across the lipid bilayer) from simulations of **T** and **O-AP** models on the *μ*s time scale are robust regarding the two tested popular force fields (AMBER99SB∗/Slipids and CHARMM36m/CHARMM36 lipid force field). Moreover, the observed trends of aggregate stability and extent of polar defects for membrane-embedded **T** (with and without *β*1-strand), **O-P,** and **O-AP**, as well as both *β*-barrel structure models are independent of the force field used. Our simulations predict that the protonation state of lysine side-chains at position 16 from the *β*1-strand influences the aggregate conformations, consistent with experimental evidence of more stable oligomeric preparations at pH 9.0 over pH 7.4 (29, 33). In simulations of tetrameric and hexameric *β*-sandwich oligomers (Tetra-2pe-2.2.2, Hexa-2pe-2.2.4), the CHARMM force field reproducibly favors aggregate reorganization towards structures that do not form pores in contrast to AMBER simulations. Here, the extent of pore formation and insertion depth of the HHQK domain is sensitive to the employed combinations of protein, lipid, and water force field in simulations. Theoretical work on plain lipid bilayers has highlighted barrier height differences for the formation of aqueous pores in MD simulations with respect to the applied force field, *i.e.* for moving a hydrophilic phospholipid headgroup to the hydrophobic bilayer center (39, 64). A potential overstabilization of the bilayer state (64), as well as shortcomings of additive MD force fields to describe ion-induced membrane defects as a function of bilayer thickness (65), therefore may explain the observed differences for the smaller, presumably metastable, oligomers models. The observed slow conformational changes furthermore underscore the need for MD simulations beyond the hundreds of nanosecond time scale to properly asses the kinetic stability of membrane-embedded *β*-sheet oligomers (48).

Based on our investigation of multiple low molecular-weight A*β* oligomer models, we surmise that hydrophilic edge pore formation induced by 6RHY type structures is compatible with a wide range of experimental observations. It has recently been suggested that toxic A*β* oligomers can be defined by structural constraints, however, are not necessarily of the same size (30, 33, 66). It is therefore intriguing to speculate that toxic *β*PFOs are not necessarily of one particular oligomer size but rather tied to the presence of layered *β*-sheet edges formed by A*β*′s N-terminal *β*-strands, stably inserted in the membrane. We have shown that this structural motif plays a key role in both, the A*β* lipid membrane interactions and characteristics of ion permeation.

Finally, the exploration of structural and functional features of membrane-inserted A*β*42 oligomers as outlined above provides an intriguing working model to further the development of therapeutic intervention strategies. Unraveling and understanding the molecular basis of aggregate-induced membrane permeabilization and cytotoxicity may assist future structure-based inhibitor design. Along these lines, the layered *β*-sheet edge conformations of A*β*42 oligomers bearing the N-terminal HHQK domain provide an intriguing target. Monoclonal antibodies (22, 23, 67) that recognize the side-by-side pair edge motif of *β*1-strands or specific regions in the N-terminus of A*β*42 could have the potential to neutralize oligomer-induced pore formation and ion permeation. A number of small molecules with specific binding properties to either exposed backbone epitopes (68, 69), histidine (58), or lysine (70) side-chains have been shown to effectively modulate A*β*42 oligomerization and to interfere with A*β*42 pore formation. Capping the formation of membrane-embedded A*β* oligomers with layered *β*-sheet conformations could be explored as another strategy to block pore-forming A*β*42 oligomers (71). The present results offer the opportunity to revisit and confer the mode of action for these and other known A*β* oligomer inhibitors (7, 67, 72, 73, 74) at the molecular level. In order to achieve enhanced efficacy, the ability to bind near or in the cellular membrane is expected to be an important property of drugs targeting A*β*42 *β*PFOs.

## Experimental procedures

### Simulation details

The GROMACS 2018 and 2020 simulation software package (75, 76) was used to set up, carry out, and analyze the MD simulations. Settings for production runs were chosen as follows: the long range electrostatic interactions were treated using the Particle Mesh Ewald method (77, 78). Bonds in protein and lipid molecules were constrained using the P-LINCS (79) algorithm. Water molecules were constrained using SETTLE (80) algorithm. Neighbor lists were updated with the Verlet list scheme (76, 81). For production runs, the simulated systems were kept at a temperature of 300 K by applying the velocity-rescaling (82) algorithm. Initial velocities for the production runs were taken according to the Maxwell-Boltzmann distribution at 300 K. The pressure was held constant by using the Parrinello-Rahman barostat (83) with a semi-isotropic coupling in the xy-plane.

### Simulation protocol: AMBER99SB∗/Slipids force field

For all simulations with the AMBER99SB^∗^-ILDN (84, 85, 86) force field, we employed the TIP3P water model (87) together with ion parameters by Dang *et al*. (88, 89). For the description of the lipids, a modified version (90) of the all-atom Slipids force field (91, 92, 93) was used. Bonds between all atoms in protein and lipid molecules were constrained. The conversion of aliphatic hydrogen atoms to virtual sites (94) in all protein and lipid molecules allowed to set the integration time step to 4 fs to speed up the simulations. A real space cut-off for the electrostatic interactions was set at 1.0 nm. The van-der-Waals interactions were cut off at 1.0 nm. A dispersion correction for energy and pressure was applied.

### Simulation protocol: CHARMM36m/CHARMM36 lipid force field

All simulations with the CHARMM36m (95, 96) protein force field utilized the CHARMM36 lipid parameters (97) together with the CHARMM-modified (98) TIP3P water model. The integration time step was set to 2 fs. The neighbor lists for nonbonded interactions were updated every 20 steps. Real-space electrostatic interactions were truncated at 1.2 nm. The van der Waals interactions were switched off between 1.0 to 1.2 nm and short-range electrostatic interactions were cut-off at 1.2 nm.

### System preparation and molecular modeling

#### A*β*(6–42) *β*-sheet and *β*-sandwich models (6RHY fold)

If not stated otherwise, all simulations of oligomeric amyloid-*β* peptide aggregate structures were initiated from or generated based on atomic coordinates from previously published solid-state NMR spectroscopy and CCS data (33). Octameric *β*-sandwich structures were derived by simple symmetry operations (translation and/or rotations) of the tetrameric *β*-sheet conformation (PDB ID: 6RHY) using the PyMOL visualization software (99, http://www.pymol.org/pymol). In the majority of the simulations, the disordered, solvent-exposed N-terminal regions that could not be assigned or did not show lipid interactions experimentally (33) were truncated. And the N-termini were capped with acetyl groups. Smaller oligomer models and aggregate isoforms were obtained by selectively removing individual A*β* molecules from the common structure templates (tetrameric *β*-sheet, octameric *β*-sandwich). F19G; H13A, H14A, Q15A, and K16N mutants were generated by substituting individual residue side-chains in the WT 6RHY model conformation using the ’Mutagenesis’ functionality PyMOL.

In addition, two distinct tetrameric and hexameric A*β β*-barrel model structures were simulated. **A***β***(6–42)** *β***-barrel model (2OTK fold):** This structure model was generated analogous to the protocol described by Nowick *et al*. (100) using atomic coordinates of X-ray crystallographic structures formed by macrocyclic mimics of the A*β*(16–36) *β*-hairpin (PDB ID: 5W4J) (38). After deletion of the delta-linked ornithine residues from each macrocycle using PyMOL, an oligomer structure with resolved A*β*(16–22 and 30–36) *β*-strands in each monomer were obtained. The previously reported monomeric *β*-hairpin structure of A*β*(16–40) (PDB ID: 2OTK) (56) with the same intramolecular hydrogen bond register was fitted on the structural template (5W4J), resulting in a combined model of a high-resolution A*β*(16–40) tetramer. Missing N- and C-terminal residues were modeled in random coil conformation with PyMOL (Fig. S7). **A***β***(21-42)** *β***-barrel model:** This structure model was generated analogous to the protocol described by Do *et al*. (37). Threading of the A*β* (28–38) sequence onto the backbone structure of the hexameric *α*B-crystallin cylindrin revealed favorable side-chain packing among several tested C-terminal A*β* fragments (37). Thus, the side-chains in the X-ray crystal structures of the *α*B-crystallin cylindrin hexamer (PDB ID: 3SGO) were replaced to match the A*β*(28–38) amino acid stretch using PyMOL.

Missing N- and C-terminal residues were modeled in random coil conformation with PyMOL (Fig. S7). After the modeling stage, both structures were equilibrated in a water box to allow structure relaxation before arriving at the final models.

In all simulation systems (see Table S2), the titratable amino acids were protonated according to their standard protonation states at pH 7, while also taking into account the solvent exposure and electrostatic interactions with neighboring polar groups. Thus, aspartic and glutamic side-chains were simulated with negative charge and all histidine side-chains were set to neutral (33, 101). All lysine side-chains were simulated as positively charged (LysH^+^). In three additional sets of simulations of *β*-sandwich models with the 6RHY fold, the lysine residues at position 16 were also simulated in neutral state (K16/LysH^0^, see Table S2).

All production runs were preceded by a multistep equilibration of the system. First, the protein part was separately energy minimized in water. Second, the aggregates were immersed in a phospholipid bilayer. Depending on the simulated oligomer model, the following two sizes of POPC membrane patches were used: a lipid bilayer composed of (1) 250 (used for **O-AP** and **O-P** *β*-sandwich models and the full-length A*β* tetramer) or (2) 170 (used for all other oligomer models) POPC lipid molecules spanning the xy-plane of the periodic simulation box (see Fig. S1). Both membrane patches were prepared with a water slab of 2.5 nm thickness on top and bottom of the bilayer using the CHARMM-GUI membrane builder webserver (102). The simulations with full-length A*β* molecules were set up with a 6.0 nm thick water slab on top and bottom of the bilayer. Lipid-solvent contacts were equilibrated for 1 ns at 300 K. Next, the *β*-sheet structure was embedded into the solvated lipid bilayer. Subsequently, K^+^ and Cl^−^ ions (ionic strength: 600 mM) were added in the aqueous phase. The whole system was simulated for an additional 1 ns. Position restraints with a force constant of 1000 kJ mol^−1^ nm^−2^ were applied to the heavy atoms of the protein backbone to allow relaxation of protein-solvent and protein-lipid contacts. Reference simulations without inserted *β*-sheet aggregates were carried out with a pure POPC membrane patch composed of 170 lipid molecules. Depending on the two types of initial bilayer sizes, the entire solvated systems consisted of roughly 42,000 to 67,000 atoms (Table S1).

### Analysis protocol

From the individual simulation trajectories, samples were collected every 100 ps and used throughout subsequent analyses. The first 500 ns of each trajectory were not considered for analyses to ensure that the results are not biased by the initial equilibration of the simulation system. The extent of the pore formation process was quantified by partial density profiles across the lipid bilayer and the occurence of continuous polar transmembrane defects.

#### Partial density profiles

The partial densities of water molecules, polar lipid groups and ions across the simulation box and along the membrane normal direction were computed using the gmx density tool. Histogram binning was done relative to the center of all lipid atoms. Partial density profiles were averaged over all independent trajectory replicates per simulation system.

#### Reaction coordinate for polar transmembrane defects

To monitor and quantify polar transmembrane defects in the model membrane, *i.e.* the presence of water molecules and the polar lipid head groups in the hydrophobic center of the phospholipid bilayer, a reaction coordinate analysis was carried out based on the work by Awasthi *et al*. (39). The reaction coordinate was defined as the parameter *ξ* and indicates the fraction of slices along the 2.8 nm thick transmembrane region that are occupied by at least one lipid or water oxygen atom in each frame. In total, 28 equally distributed slices along the membrane normal with 0.1 nm increments were used. A value of 0 for *ξ* thus means that no slice in the hydrophobic transmembrane slab is occupied by polar atoms. A value of 1 for *ξ* means that all slices are occupied by polar atoms, therefore indicating a continuous polar defect in the membrane. Reaction coordinate values for each simulation frame from all independent trajectory replicates per simulation system were combined, sorted into 1-d bins and normalized. The probability *p*_*i*_ = *ξ*_*i,j*_*/ξ*_*total*_ was calculated, where *ξ*_*i,j*_ is the number of frames *j* in the bin *i* and *ξ*_*total*_ is the total number of frames. The logarithm of *p*(*ξ*_*i*_) multiplied by a constant factor (-RT, with T = 300 K) results in a free energy value in units of kJ/mol.

#### Contact analysis and mapping

The frequency of short-range interatomic contacts between the peptide oligomer structures and water, polar lipid groups, as well as ions was quantified for every frame using the contact search algorithm provided by the g contacts program (103). Pairwise residue-based contacts with a cutoff distance of 0.5 nm between heavy atoms of the analyzed groups were averaged over all independent trajectory replicates per simulation system.

#### RMSD of atomic positions

Comparison of oligomer model conformations obtained by the MD simulations with respect to the starting structure by calculating rmsd of mainchain and C_*β*_ atoms from the (transmembrane) *β*-strands.

#### Visualization

Renderings of atomic coordinates were carried out with the molecular visualization software PyMOL (99).

## Data availability

MD data generated during this study and source data for this paper are deposited in the open research data repository Edmond at https://doi.org/10.17617/3.UR3ECA. Additional raw MD data generated in this study are available from the corresponding author upon reasonable request.

## Supporting information

This article contains supporting information.

## Conflict of interests

The authors declare that they have no conflicts of interest with the contents of this article.

## Supporting information

Movie-4A

Movie-8B

movie-8BPERM

## References

[1] Hardy J., Selkoe D.J. (2002). The amyloid hypothesis of alzheimer’s disease: progress and problems on the road to therapeutics,. Science.

[2] Yankner B.A., Lu T. (2009). Amyloid *β*-protein toxicity and the pathogenesis of alzheimer disease. J. Biol. Chem..

[3] Sakono M., Zako T. (2010). Amyloid oligomers: formation and toxicity of a*β* oligomers. FEBS J..

[4] Knowles T.P.J., Vendruscolo M., Dobson C.M. (2014). The amyloid state and its association with protein misfolding diseases. Nat. Rev. Mol. Cell Biol..

[5] Selkoe D.J., Hardy J. (2016). The amyloid hypothesis of alzheimer’s disease at 25 years. EMBO Mol. Med..

[6] Pannuzzo M. (2021). Beta-amyloid pore linked to controlled calcium influx into the cell: a new paradigm for alzheimer’s disease. Alzheimer’s Demen..

[7] Chen G.-f., Xu T.-h., Yan Y., Zhou Y.-r., Jiang Y., Melcher K. (2017). Amyloid beta: structure, biology and structure-based therapeutic development. Acta Pharmacol. Sin..

[8] Viola K.L., Klein W.L. (2015). Amyloid *β* oligomers in alzheimer’s disease pathogenesis, treatment, and diagnosis. Acta Neuropathol..

[9] Cleary J.P., Walsh D.M., Hofmeister J.J., Shankar G.M., Kuskowski M.A., Selkoe D.J. (2004). Natural oligomers of the amyloid-*β* protein specifically disrupt cognitive function. Nat. Neurosci..

[10] De Strooper B., Karran E. (2016). The cellular phase of Alzheimer’s disease. Cell.

[11] De S., Whiten D.R., Ruggeri F.S., Hughes C., Rodrigues M., Sideris D.I. (2019). Soluble aggregates present in cerebrospinal fluid change in size and mechanism of toxicity during Alzheimer’s disease progression. Acta Neuropathol. Commun..

[12] Shankar G.M., Li S., Mehta T.H., Garcia-Munoz A., Shepardson N.E., Smith I. (2008). Amyloid-*β* protein dimers isolated directly from Alzheimer^’^s brains impair synaptic plasticity and memory. Nat. Med..

[13] Brinkmalm G., Hong W., Wang Z., Liu W., O’Malley T.T., Sun X. (2019). Identification of neurotoxic cross-linked amyloid-*β* dimers in the Alzheimer’s brain. Brain.

[14] Giulian D., Haverkamp L.J., Yu J., Karshin W., Tom D., Li J. (1998). The hhqk domain of *β*-amyloid provides a structural basis for the immunopathology of Alzheimer’s disease. J. Biol. Chem..

[15] Lambert M.P., Barlow A.K., Chromy B.A., Edwards C., Freed R., Liosatos M. (1998). Diffusible, nonfibrillar ligands derived from a*β* 1–42 are potent central nervous system neurotoxins. Proc. Natl. Acad. Sci. U. S. A..

[16] Kayed R., Head E., Thompson J.L., McIntire T.M., Milton S.C., Cotman C.W. (2003). Common structure of soluble amyloid oligomers implies common mechanism of pathogenesis. Science.

[17] Butterfield S.M., Lashuel H.A. (2010). Amyloidogenic protein-membrane interactions: mechanistic insight from model systems. Angewandte Chemie Internat. Ed.n.

[18] Zhang Y.-J., Shi J.-M., Bai C.-J., Wang H., Li H.-Y., Wu Y. (2012). Intra-membrane oligomerization and extra-membrane oligomerization of amyloid-*β* peptide are competing processes as a result of distinct patterns of motif interplay. J. Biol. Chem..

[19] Jana M.K., Cappai R., Pham C.L.L., Ciccotosto G.D. (2016). Membrane-bound tetramer and trimer a*β* oligomeric species correlate with toxicity towards cultured neurons. J. Neurochem..

[20] De S., Wirthensohn D.C., Flagmeier P., Hughes C., Aprile F.A., Ruggeri F.S. (2019). Different soluble aggregates of a*β*42 can give rise to cellular toxicity through different mechanisms. Nat. Commun..

[21] Bode D.C., Freeley M., Nield J., Palma M., Viles J.H. (2019). Amyloid-*β* oligomers have a profound detergent-like effect on lipid membrane bilayers, imaged by atomic force and electron microscopy. J. Biol. Chem..

[22] Dunys J., Valverde A., Checler F. (2018). Are n- and c-terminally truncated a*β* species key pathological triggers in Alzheimer’s disease?. J. Biol. Chem..

[23] Karkisaval A.G., Rostagno A., Azimov R., Ban D.K., Ghiso J., Kagan B.L. (2020). Ion channel formation by n-terminally truncated a*β* (4-42): relevance for the pathogenesis of Alzheimer’s disease, nanomedicine: nanotechnology. Biol. Med..

[24] Arispe N., Rojas E., Pollard H.B. (1993). Alzheimer disease amyloid beta protein forms calcium channels in bilayer membranes: blockade by tromethamine and aluminum.. Proc. Natl. Acad. Sci. U. S. A..

[25] Quist A., Doudevski I., Lin H., Azimova R., Ng D., Frangione B. (2005). Amyloid ion channels: a common structural link for protein-misfolding disease. Proc. Natl. Acad. Sci. U. S. A..

[26] Bode D.C., Baker M.D., Viles J.H. (2017). Ion channel formation by amyloid-*β*42 oligomers but not amyloid-*β*40 in cellular membranes. J. Biol. Chem..

[27] Caughey B., Lansbury P.T. (2003). Protofibrils, pores, fibrils, and neurodegeneration: separating the responsible protein aggregates from the innocent bystanders. Annu. Rev. Neurosci..

[28] Lashuel H.A., Lansbury P.T. (2006). Are amyloid diseases caused by protein aggregates that mimic bacterial pore-forming toxins?. Q. Rev. Biophys..

[29] Serra-Batiste M., Ninot-Pedrosa M., Bayoumi M., Gair'ı M., Maglia G., Carulla N. (2016). A*β*42 assembles into specific *β*-barrel pore-forming oligomers in membrane-mimicking environments. Proc. Natl. Acad. Sci. U. S. A..

[30] Osterlund N., Moons R., Ilag L.L., Sobott F., Gräslund A. (2019). Native ion mobility-mass spectrometry reveals the formation of *β*-barrel shaped amyloid-*β* hexamers in a membrane-mimicking environment. J. Am. Chem. Soc..

[31] Hirakura Y., Lin M.-C., Kagan B.L. (1999). Alzheimer amyloid a*β*1-42 channels: effects of solvent, ph, and Congo red. J. Neurosci. Res..

[32] Demuro A., Parker I. (2013). Cytotoxicity of intracellular a*β*42 amyloid oligomers involves ca2+ release from the endoplasmic reticulum by stimulated production of inositol trisphosphate. J. Neurosci..

[33] Ciudad S., Puig E., Botzanowski T., Meigooni M., Arango A.S., Do J. (2020). A*β*(1-42) tetramer and octamer structures reveal edge conductivity pores as a mechanism for membrane damage.. Nat. Commun..

[34] Samdin T.D., Kreutzer A.G., Nowick J.S. (2021). Exploring amyloid oligomers with peptide model systems. Curr. Opin. Chem. Biol..

[35] Tiiman A., Jarvet J., Gräslund A., Vukojevic V. (2015). Heterogeneity and turnover of intermediates during amyloid-*β* (a*β*) peptide aggregation studied by fluorescence correlation spectroscopy. Biochemistry.

[36] Lendel C., Bjerring M., Dubnovitsky A., Kelly R.T., Filippov A., Antzutkin O.N. (2014). A hexameric peptide barrel as building block of amyloid-*β* protofibrils. Angew. Chem. Int. Ed..

[37] Do T.D., LaPointe N.E., Nelson R., Krotee P., Hayden E.Y., Ulrich B. (2016). Amyloid *β*-protein c-terminal fragments: formation of cylindrins and *β*-barrels. J. Am. Chem. Soc..

[38] Kreutzer A.G., Spencer R.K., McKnelly K.J., H I.L., Yoo S., Salveson P.J. (2017). A hexamer of a peptide derived from aβ16-36. Biochemistry.

[39] Awasthi N., Hub J.S. (2016). Simulations of pore formation in lipid membranes: reaction coordinates, convergence, hysteresis, and finite-size effects. J. Chem. Theor. Comput..

[40] Adler J., Scheidt H.A., Krüger M., Thomas L., Huster D. (2014). Local interactions influence the fibrillation kinetics, structure and dynamics of a*β*(1-40) but leave the general fibril structure unchanged. Phys. Chem. Chem. Phys..

[41] Das A.K., Rawat A., Bhowmik D., Pandit R., Huster D., Maiti S. (2015). An early folding contact between phe19 and leu34 is critical for amyloid-*β* oligomer toxicity. ACS Chem. Neurosci..

[42] Winkler K., Scharnagl H., Tisljar U., Hoschützky H., Friedrich I., Hoffmann M.M. (1999). Competition of a*β* amyloid peptide and apolipoprotein e for receptor-mediated endocytosis. J. Lipid Res..

[43] Smith D.P., Smith D.G., Curtain C.C., Boas J.F., Pilbrow J.R., Ciccotosto G.D. (2006). Copper-mediated amyloid-*β* toxicity is associated with an intermolecular histidine bridge. J. Biol. Chem..

[44] Kaden D., Harmeier A., Weise C., Munter L.M., Althoff V., Rost B.R. (2012). Novel app/a*β* mutation k16n produces highly toxic heteromeric a*β* oligomers. EMBO Mol. Med..

[45] Lemkul J.A., Bevan D.R. (2013). Aggregation of alzheimer’s amyloid *β*-peptide in biological membranes: a molecular dynamics study. Biochemistry.

[46] Das P., Chacko A.R., Belfort G. (2016). Alzheimer’s protective cross-interaction between wild-type and a2t variants alters a*β* 42 dimer structure. ACS Chem. Neurosci..

[47] Barz B., Liao Q., Strodel B. (2017). Pathways of amyloid-*β* aggregation depend on oligomer shape. J. Am. Chem. Soc..

[48] Sepehri A., Lazaridis T. (2022). Putative structures of membrane-embedded amyloid *β* oligomers. ACS Chem. Neurosci..

[49] Nguyen P.H., Campanera J.M., Ngo S.T., Loquet A., Derreumaux P. (2019). Tetrameric a*β*40 and a*β*42 *β*-barrel structures by extensive atomistic simulations. i. in a bilayer mimicking a neuronal membrane. The J. Phys. Chem. B.

[50] Jang H., Zheng J., Nussinov R. (2007). Models of *β*-amyloid ion channels in the membrane suggest that channel formation in the bilayer is a dynamic process. Biophys. J..

[51] Xiang N., Lyu Y., Zhu X., Narsimhan G. (2018). Investigation of the interaction of amyloid *β* peptide (11–42) oligomers with a 1-palmitoyl-2-oleoyl-sn-glycero-3-phosphocholine (POPC) membrane using molecular dynamics simulation. Phys. Chem. Chem. Phys..

[52] Nguyen P.H., Ramamoorthy A., Sahoo B.R., Zheng J., Faller P., Straub J.E. (2021). Amyloid oligomers: a joint experimental/computational perspective on alzheimer’s disease, Parkinson’s disease, type II diabetes, and amyotrophic lateral sclerosis. Chem. Rev..

[53] Korshavn K.J., Satriano C., Lin Y., Zhang R., Dulchavsky M., Bhunia A. (2017). Reduced lipid bilayer thickness regulates the aggregation and cytotoxicity of amyloid-*β*. J. Biol. Chem..

[54] Bernstein S.L., Dupuis N.F., Lazo N.D., Wyttenbach T., Condron M.M., Bitan F. (2009). Amyloid-*β* protein oligomerization and the importance of tetramers and dodecamers in the aetiology of alzheimer^’^s disease. Nat. Chem..

[55] Kikuchi N., Fujiwara K., Ikeguchi M. (2018). *β*-strand twisting/bending in soluble and transmembrane *β*-barrel structures. Proteins: Structure, Function, and Bioinform..

[56] Hoyer W., Grönwall C., Jonsson A., Ståhl S., Härd T. (2008). Stabilization of a *β*-hairpin in monomeric alzheimer’s amyloid-*β* peptide inhibits amyloid formation. Proc. Natl. Acad. Sci. U. S. A..

[57] Scala C.D., Yahi N., Boutemeur S., Flores A., Rodriguez L., Chahinian H. (2016). Common molecular mechanism of amyloid pore formation by alzheimer’s *β*-amyloid peptide and *α*-synuclein. Sci. Rep..

[58] Arispe N., Diaz J.C., Flora M. (2008). Efficiency of histidine-associating compounds for blocking the alzheimer^’^s a*β* channel activity and cytotoxicity. Biophys. J..

[59] Minicozzi V., Stellato F., Comai M., Serra M.D., Potrich C., Meyer-Klaucke W. (2008). Identifying the minimal copper- and zinc-binding site sequence in amyloid-*β* peptides. J. Biol. Chem..

[60] Nair N.G., Perry G., Smith M.A., Reddy V.P. (2010). NMR studies of zinc, copper, and iron binding to histidine, the principal metal ion complexing site of amyloid-*β* peptide. J. Alzheimer^’^s Dis..

[61] Lee M.-C., Yu W.-C., Shih Y.-H., Chen C.-Y., Guo Z.-H., Huang S.-J. (2018). Zinc ion rapidly induces toxic, off-pathway amyloid-*β* oligomers distinct from amyloid-*β* derived diffusible ligands in alzheimer’s disease. Sci. Rep..

[62] Wu J., Blum T.B., Farrell D.P., DiMaio F., Abrahams J.P., Luo J. (2021). Cryo-electron microscopy imaging of alzheimer^’^s amyloid-beta 42 oligomer displayed on a functionally and structurally relevant scaffold. Angewandte Chemie Inter. Ed..

[63] Durell S.R., Kayed R., Guy H.R. (2022). The amyloid concentric *β*-barrel hypothesis: models of amyloid beta 42 oligomers and annular protofibrils. Proteins: Structure, Function, and Bioinformatics.

[64] Bennett W.F.D., Hong C.K., Wang Y., Tieleman D.P. (2016). Antimicrobial peptide simulations and the influence of force field on the free energy for pore formation in lipid bilayers. J. Chem. Theory and Comput..

[65] Chen P., Vorobyov I., Roux B., Allen T.W. (2021). Molecular dynamics simulations based on polarizable models show that ion permeation interconverts between different mechanisms as a function of membrane thickness. J. Phys. Chem. B.

[66] Vadukul D.M., Maina M., Franklin H., Nardecchia A., Serpell L.C., Marshall K.E. (2020). Internalisation and toxicity of amyloid-*β* 1-42 are influenced by its conformation and assembly state rather than size. FEBS Lett..

[67] Shea D., Daggett V. (2022). Amyloid-*β* oligomers: multiple moving targets. Biophysica.

[68] Hernandez A.M., Urbanke H., Gillman A.L., Lee J., Ryazanov S., Agbemenyah H.Y. (2017). The diphenylpyrazole compound anle138b blocks a*β* channels and rescues disease phenotypes in a mouse model for amyloid pathology. EMBO Mol. Med..

[69] Matthes D., Gapsys V., Griesinger C., de Groot B. (2017). Resolving the atomistic modes of anle138b inhibitory action on peptide oligomer formation.. ACS Chem. Neurosci..

[70] Sinha S., Lopes D.H.J., Du Z., Pang E.S., Shanmugam A., Lomakin A. (2011). Lysine-specific molecular tweezers are broad-spectrum inhibitors of assembly and toxicity of amyloid proteins. J. Am. Chem. Soc..

[71] Fu Z., Aucoin D., Ahmed M., Ziliox M., Nostrand W.E.V., Smith S.O. (2014). Capping of a*β*42 oligomers by small molecule inhibitors. Biochemistry.

[72] Necula M., Kayed R., Milton S., Glabe C.G. (2007). Small molecule inhibitors of aggregation indicate that amyloid *β* oligomerization and fibrillization pathways are independent and distinct. J. Biol. Chem..

[73] Nie Q., guang Du X., yu Geng M. (2011). Small molecule inhibitors of amyloid *β* peptide aggregation as a potential therapeutic strategy for alzheimer’s disease. Acta PharmacoL. Sin..

[74] Puig E., Tolchard J., Riera A., Carulla N. (2020). Somatostatin, an *in vivo* binder to a*β* oligomers, binds to *β*PFOa*β*(1-42) tetramers. ACS Chem. Neurosci..

[75] Pronk S., Pande S., S.ll V., Schulz R., Larsson P., Bjelkmar P. (2013). Gromacs 4.5: a high-throughput and highly parallel open source molecular simulation toolkit. Bioinformatics.

[76] Abraham M.J., Murtola T., Schulz R., Pall S., Smith J.C., Hess B. (2015). Gromacs: high performance molecular simulations through multi-level parallelism from laptops to supercomputers. SoftwareX.

[77] Darden T., York D., Pedersen L. (1993). Particle mesh ewald: an n-log(n) method for ewald sums in large systems. J. Chem. Phys..

[78] Essmann U., Perera L., Berkowitz M.L., Darden T., Lee H., Pedersen L.G. (1995). A smooth particle mesh Ewald method,. J. Chem. Phys..

[79] Hess B. (2008). P-Lincs: a parallel linear constraint solver for molecular simulation. J. Chem. Theor. Comput..

[80] Miyamoto S., Kollman P.A. (1992). Settle: an analytical version of the shake and rattle algorithm for rigid water models. J. Comput. Chem..

[81] Verlet L. (1967). Computer ”experiments” on classical fluids. i. thermodynamical properties of Lennard-Jones molecules. Phys. Rev..

[82] Bussi G., Donadio D., Parrinello M. (2007). Canonical sampling through velocity rescaling. J. Chem. Phys..

[83] Parrinello M., Rahman A. (1981). Polymorphic transitions in single crystals: a new molecular dynamics method,. J. Appl. Phys..

[84] Hornak V., Abel R., Okur A., Strockbine B., Roitberg A., Simmerling C. (2006). Comparison of multiple amber force fields and development of improved protein backbone parameters. Proteins: Struct. Funct. Bioinf..

[85] Best R.B., Hummer G. (2009). Optimized molecular dynamics force fields applied to the helix-coil transition of polypeptides.. J. Phys. Chem. B.

[86] Lindorff-Larsen K., Piana S., Palmo K., Maragakis P., Klepeis J.L., Dror R.O. (2010). Improved side-chain torsion potentials for the amber ff99sb protein force field. Proteins: Struct. Funct. Bioinf..

[87] Jorgensen W.L., Chandrasekhar J., Madura J.D., Impey R.W., Klein M.L. (1983). Comparison of simple potential functions for simulating liquid water.. J. Chem. Phys..

[88] Smith D.E., Dang L.X. (1994). Computer simulations of nacl association in polarizable water. J. Chem. Phys..

[89] Chang T.-M., Dang L.X. (1999). Detailed study of potassium solvation using molecular dynamics techniques. J. Phys. Chem. B.

[90] Melcr J., Bonhenry D., Timr S., Jungwirth P. (2016). Transmembrane potential modeling: comparison between methods of constant electric field and ion imbalance. J. Chem. Theor. Comput..

[91] Jämbeck J.P.M., Lyubartsev A.P. (2012). An extension and further validation of an all-atomistic force field for biological membranes. J. Chem. Theor. Comput..

[92] Jämbeck J.P.M., Lyubartsev A.P. (2012). Derivation and systematic validation of a refined all-atom force field for phosphatidylcholine lipids. J. Phys. Chem. B.

[93] Jämbeck J.P.M., Lyubartsev A.P. (2013). Another piece of the membrane puzzle: extending slipids further. J. Chem. Theor. Comput..

[94] Feenstra K.A., Hess B., Berendsen H.J.C. (1999). Improving efficiency of large time-scale molecular dynamics simulations of hydrogen-rich systems. J. Comput. Chem..

[95] Best R.B., Zhu X., Shim J., Lopes P.E.M., Mittal J., Feig M. (2012). Optimization of the additive charmm all-atom protein force field targeting improved sampling of the backbone phi, psi and side-chain chi1 and chi2 dihedral angles. J. Chem. Theor. Comput..

[96] Huang J., Rauscher S., Nawrocki G., Ran T., Feig M., de Groot B.L. (2017). Charmm36m: an improved force field for foldedand intrinsically disordered proteins. Nat. Met..

[97] Klauda J.B., Venable R.M., Freites J.A., O Connor J.W., Tobias D.J., Mondragon-Ramirez C. (2010). Update of the charmm all-atom additive force field for lipids: validation on six lipid types. J. Phys. Chem. B.

[98] MacKerell A.D., Bashford D., Bellott M., Dunbrack R.L., Evanseck J.D., Field M.J. (1998). All-atom empirical potential for molecular modeling and dynamics studies of proteins. J. Phys. Chem. B.

[99] Schrödinger L., DeLano W. (2020).

[100] Salveson P.J., Spencer R.K., Nowick J.S. (2016). X-ray crystallographic structure of oligomers formed by a toxic *β*-hairpin derived from *α*-synuclein: trimers and higher-order oligomers. J. Am. Chem. Soc..

[101] Teixeira V.H., Vila-Vicosa D., Reis P.B.P.S., Machuqueiro M. (2016). Pka values of titrable amino acids at the water/membrane interface. J. Chem. Theor. Comput..

[102] Jo S., Kim T., Iyer V.G., Im W. (2008). Charmm-gui: a web-based graphical user interface for charmm,. J. Comput. Chem..

[103] Blau C., Grubmüller H. (2013). g contacts: fast contact search in bio-molecular ensemble data. Computer Phys. Commun..

