# Supplementary material for: Molecular dynamics simulations reveal the importance of amyloid-beta oligomer β-sheet edge conformations in membrane permeabilization
Source: J Biol Chem. 2023 Feb 16;299(4):103034. doi: 10.1016/j.jbc.2023.103034 (PMC10033322; doi:10.1016/j.jbc.2023.103034)

**Supplementary Movie Legends**

**Supporting Movie S1. Formation of a partial polar defect in a POPC bilayer induced by an Aβ42 β-sheet tetramer.** The movie shows the first 75 ns of a representative MD trajectory for a tetrameric Aβ42 βPFO with the 6RHY fold embedded in a phospholipid bilayer. The oligomer model is shown in transparent surface and cartoon representation. The β-strands of the transmembrane domain are colored as follows: residues G9-A21 (β1, cool-yellow); residues G29-V40 (β2, light blue); residues G29-I41 (β3, green). Side-chain atoms of residues H13-K16 from the β1 edge strand are shown with sticks. Lipid phosphate atoms are shown as spheres. For clarity, ions, water and aliphatic chains of the lipid molecules were omitted.

**Supporting Movie S2. Formation of a continuous polar defect in a POPC bilayer induced by an Aβ42 β-sandwich octamer.** The movie shows the first 75 ns of a representative MD trajectory for an octameric Aβ42 βPFO with the 6RHY fold embedded in a phospholipid bilayer. The oligomer model is shown in transparent surface and cartoon representation. The β-strands of the transmembrane domain are colored as follows: residues G9-A21 (β1, cool-yellow); residues G29-V40 (β2, light-blue); residues G29-I41 (β3, green). Side-chain atoms of residues H13-K16 from the β1 edge strand are shown with sticks. Lipid phosphate atoms are shown as spheres. For clarity, ions, water and aliphatic chains of the lipid molecules were omitted.

**Supporting Movie S3. Ion permeability of a phospholipid membrane is facilitated by direct interactions with β1-strand edges.** The movie shows 100 ns of a potassium ion permeation event from a representative MD trajectory. For clarity, other ions, as well as all water and lipid molecules were omitted and only the membrane embedded octameric β-sandwich edge is shown simultaneously from the side of the β-sheets (left) and along the direction of the backbone hydrogen bonds of the β-sheets (right). The scence is centered on the β1-strand of the oligomer model which is shown in transparent surface and cartoon representation. Residues G9-A21 from the β1-strand are represented as sticks. Solvent-facing atoms from the protein backbone (residues V12-F20) and side-chain atoms of residues H13-K16 are highlighted by wider sticks. The permeating potassium ion is shown as sphere. For clarity, other ions, all water and lipid molecules were omitted.

**Supplementary Table S1.** Summary of simulated systems.

| **System** | **no. of atoms** | **no. of waters** | **no. of ions** | **no. of POPC lipids** |
| --- | --- | --- | --- | --- |
| Tetramer, full-length Aβ42 | 138115 | 34695 | 527 K^+^, 515 Cl^−^ | 210 |
| Tetramer (T) | 44076 | 7090 | 169 K^+^, 159 Cl^−^ | 143 |
| Octamer (O-AP) | 67622 | 11178 | 264 K^+^, 244 Cl^−^ | 207 |

**Supplementary Table S2.** Summary of simulated systems and simulation data. For each A*β* oligomer, a unique identifier is given and listed together with the total number of independent MD simulation replicates, including the length of individual trajectories.

| **Aβ42 oligomer system** | | | **AMBER** | **CHARMM** |
| --- | --- | --- | --- | --- |
| Size (No. of β-strands, structure) | PDB template | Identifier | No. of sim. | No. of sim. |
| with at least one side-by-side β-strand pair edge (β1-strands) | | | | |
| Octamer (12, β-sandwich) | 6RHY | Octa-2pe.4.4.4 | 10 x 5.0 µs | 10 x 2.5 µs |
| Octamer (12, β-sandwich) | 6RHY | Octa-2pe.4.4.4 (K16/Lys^0^) | 7 x 2.5 µs | 7 x 2.5 µs |
| Octamer (12, β-sandwich) | 6RHY | Octa-2pe.4.4.4 (face-to-back) | 10 x 2.5 µs | 10 x 2.5 µs |
| Hexamer (9, β-sandwich) | 6RHY | Hexa-1pe.3.3.3 | 10 x 5.0 µs | 10 x 2.5 µs |
| Hexamer (9, β-sandwich) | 6RHY | Hexa-1pe.3.3.3 (K16/Lys^0^) | 7 x 2.5 µs | 7 x 2.5 µs |
| Hexamer (8, β-sandwich) | 6RHY | Hexa-2pe.2.2.4 | 10 x 5.0 µs | 10 x 2.5 µs |
| Hexamer (8, β-sandwich) | 6RHY | Hexa-2pe.2.2.4 (F19G) | 7 x 5.0 µs | - |
| Hexamer (8, β-sandwich) | 6RHY | Hexa-2pe.2.2.4 (H1314A,Q15A) | 7 x 5.0 µs | - |
| Hexamer (8, β-sandwich) | 6RHY | Hexa-2pe.2.2.4 (K16N) | 7 x 5.0 µs | - |
| Pentamer (8, β-sandwich) | 6RHY | Penta-1pe.3.3.2 | 10 x 5.0 µs | - |
| Pentamer (7, β-sandwich) | 6RHY | Penta-1pe.2.2.3 | 10 x 5.0 µs | - |
| Tetramer (6, β-sandwich) | 6RHY | Tetra-2pe.2.2.2 | 10 x 5.0 µs | 10 x 2.5 µs |
| Tetramer (6, β-sandwich) | 6RHY | Tetra-2pe.2.2.2 (K16/Lys^0^) | 7 x 2.5 µs | 7 x 2.5 µs |
| Tetramer (6, β-sandwich) | 6RHY | Tetra-2pe.2.2.2 (from AMBER) | - | 5 x 2.0 µs |
| Tetramer (6, β-sandwich) | 6RHY | Tetra-2pe.2.2.2 (w/o lipids) | 5 x 1.0 µs | 5 x 1.0 µs |
| Tetramer (6, β-sandwich) | 6RHY | Tetra-1pe.2.2.2 | 7 x 5.0 µs | - |
| Trimer (4, β-sandwich) | 6RHY | Tri-1pe.2.2.1 | 7 x 5.0 µs | - |
| Dimer (4, β-sandwich) | 6RHY | Di-1pe.2.2.0 | 7 x 5.0 µs | - |
| with side-by-side β-strand pair edges (β2/3-strands) | | | | |
| Octamer (8, β-sandwich) | 6RHY | Octa-2pe.0.4.4 | 10 x 5.0 µs | - |
| Hexamer (6, β-sandwich) | 6RHY | Hexa-2pe.0.2.4 | 10 x 5.0 µs | - |
| Tetramer (4, β-sandwich) | 6RHY | Tetra-2pe.0.2.2 | 7 x 5.0 µs | - |
| Tetramer (4, β-sandwich) | 6RHY | Tetra-2pe.0.0.4 | 10 x 5.0 µs | - |
| with single β-strand edges | | | | |
| Octamer (10, β-sandwich) | 6RHY | Octa-0pe.2.4.4 | 7 x 5.0 µs | - |
| Hexamer (8, β-sandwich) | 6RHY | Hexa-0pe.2.2.4 | 10 x 5.0 µs | - |
| Hexamer (6, β-sandwich) | 6RHY | Hexa-0pe.0.2.4 | 7 x 5.0 µs | - |
| Tetramer (6, β-sheet) | 6RHY | Tetra-0pe.2.2.2 (full-length Aβ42) | 7 x 1.0 µs | - |
| Tetramer (6, β-sheet) | 6RHY | Tetra-0pe.2.2.2 | 10 x 5.0 µs | 10 x 2.5 µs |
| Tetramer (6, β-sheet) | 6RHY | Tetra-0pe.2.2.2 (w/o lipids) | 5 x 1.0 µs | 5 x 1.0 µs |
| Tetramer (4, β-sheet) | 6RHY | Tetra-0pe.0.2.2 | 7 x 5.0 µs | 7 x 2.5 µs |
| Trimer (4, β-sheet) | 6RHY | Tri-0pe.1.1.2 | 7 x 5.0 µs | - |
| with no β-strand edge | | | | |
| Hexamer (6, β-barrel) | 3SGO | Hexa-0pe.3SGO | 7 x 5.0 µs | 5 x 2.5 µs |
| Hexamer (6, β-barrel) | 3SGO | Hexa-0pe.3SGO (w/o lipids) | 5 x 1.0 µs | 5 x 1.0 µs |
| Tetramer (8, β-barrel) | 5W4J/2OTK | Tetra-0pe.2OTK | 5 x 5.0 µs | 5 x 2.5 µs |
| Tetramer (8, β-barrel) | 5W4J/2OTK | Tetra-0pe.2OTK (w/o lipids) | 5 x 1.0 µs | 5 x 1.0 µs |
| pure POPC bilayer without aggregates | | | | |
| - | - | - | 3 x 1.0 µs | 3 x 1.0 µs |

**Supplementary Table S3.** Reference of simulated systems. The table lists each simulation system and the figure(s) where the respective simulation data was used for analysis.

| **Aβ42 oligomer system** | **Figure(s)** |
| --- | --- |
| Identifier |  |
| with at least one side-by-side β-strand pair edge (β1-strands) | |
| Octa-2pe.4.4.4 | 1; 2; 3; 4; 5; 8; 9; S1; S3; S5; S6; S10; S11 |
| Octa-2pe.4.4.4 (K16/Lys^0^) | 8; 9; S10; S11 |
| Octa-2pe.4.4.4 (face-to-back) | 1; 2; 3 |
| Hexa-1pe.3.3.3 | 4; 8; 9; S11 |
| Hexa-1pe.3.3.3 (K16/Lys^0^) | 8; 9; S11 |
| Hexa-2pe.2.2.4 | 4; 5; 8; 9; 10 |
| Hexa-2pe.2.2.4 (F19G) | 10 |
| Hexa-2pe.2.2.4 (H1314A,Q15A) | 10 |
| Hexa-2pe.2.2.4 (K16N) | 10 |
| Penta-1pe.3.3.2 | 4; 8 |
| Penta-1pe.2.2.3 | 4; 8 |
| Tetra-2pe.2.2.2 | 4; 5; 8; 7; 9; 10; S11; S12 |
| Tetra-2pe.2.2.2 (K16/Lys^0^) | 8; 9; S11 |
| Tetra-2pe.2.2.2 (from AMBER) | S12 |
| Tetra-2pe.2.2.2 (w/o lipids) | 7 |
| Tetra-1pe.2.2.2 | 4; 8 |
| Tri-1pe.2.2.1 | 4; 8 |
| Di-1pe.2.2.0 | 4; 8 |
| with side-by-side β-strand pair edges (β2/3-strands) | |
| Octa-2pe.0.4.4 | 4; 5 |
| Hexa-2pe.0.2.4 | 4; 5 |
| Tetra-2pe.0.2.2 | 4; 5; 8 |
| Tetra-2pe.0.0.4 | 4; 5; 8 |
| with single β-strand edges | |
| Octa-0pe.2.4.4 | 4; 6 |
| Hexa-0pe.2.2.4 | 4; 6 |
| Hexa-0pe.0.2.4 | 4; 6 |
| Tetra-0pe.2.2.2 (full-length Aβ42) | 1; S1; S2 |
| Tetra-0pe.2.2.2 | 1; 2; 3; 4; 6; 7; S1; S2; S3; S4 |
| Tetra-0pe.2.2.2 (w/o lipids) | 7 |
| Tetra-0pe.0.2.2 | 4; 6 |
| Tri-0pe.1.1.2 | 4; 6 |
| with no β-strand edge | |
| Hexa-0pe.3SGO | 6; 7; S7; S8; S9 |
| Hexa-0pe.3SGO (w/o lipids) | 7 |
| Tetra-0pe.2OTK | 6; 7; S7; S8; S9 |
| Tetra-0pe.2OTK (w/o lipids) | 7 |

**Supplementary Figure Legends**

**Figure S1. Simulation systems to study Aβ42 pore-forming oligomers.** Starting structure of the tetrameric β-sheet with **A** full-length and **B** N-terminally truncated Aβ42 molecules, as well as **C** octameric β-sandwich oligomer model immersed in a POPC bilayer. For clarity, the simulation boxes are shown once without water and ions (left) and again with explicit solvent environment (right), respectively.

**Figure S2. Polar transmembrane defects induced by full-length and N-terminally truncated Aβ42 pore-forming tetramers.** **A** Partial density profiles for water, polar lipid groups, potassium and chloride ions are shown for full-length (left) and N-terminally truncated (right) Aβ42 pore-forming β-sheet tetramers with the 6RHY fold. **B** Occurrence of a continuous polar defect across the POPC bilayer. Data are averaged over multiple independent simulations, shading indicates the standard error. As reference, profiles and distributions for an unperturbed lipid bilayer are also reported (broken lines). **C** Snapshots from a representative simulation are shown in cartoon rendering for full-length Aβ42 tetramer. Lipid phosphate atoms are shown as orange spheres.

**Figure S3. Convergence of polar transmembrane defects induced by Aβ42 pore-forming oligomers on the µs time scale.** **A** Partial density profiles for water, polar lipid groups, potassium and chloride ions are shown for the T (left) and O-AP Aβ42 oligomer models as a function of individual trajectory length: 2.5 µs (continuous lines) versus 5.0 µs (dotted lines). **B** Occurrence of a continuous polar defect across the POPC bilayer. Data are averaged over multiple independent simulations, shading indicates the standard error. As reference, profiles and distributions for an unperturbed lipid bilayer are also reported (broken lines).

**Figure S4. Detailed contact maps for the Aβ42 tetrameric β-sheet oligomer model.** Averaged contact frequencies of direct contacts between **A** water molecules and **B** polar lipid groups to protein atoms mapped onto the tetrameric β-sheet structure (T) for two tested force fields (AMBER, CHARMM) shown in cartoon representation. Side-chain atoms for residues 13-16 and 35 are shown in stick representation. **C** Detailed breakdown of average contact frequencies for residues 12-20 from the β1-strand edge to several POPC lipid groups, water and potassium ions are compared for the two tested force fields. The standard error is indicated by error bars.

**Figure S5. Detailed contact maps for the Aβ42 octameric β-sandwich oligomer model.**  Averaged contact frequencies of direct contacts between **A** water molecules and **B** polar lipid groups to protein atoms mapped onto the octameric β-sandwich structure (O-AP) for two tested force fields (AMBER, CHARMM) shown in cartoon representation. Side-chain atoms for residues 13-16 and 35 are shown in stick representation. **C** Detailed breakdown of average contact frequencies for residues 12-20 from the side-by-side β1-strand pair edge to several POPC lipid groups, water and potassium ions are compared for the two tested force fields. The standard error is indicated by error bars.

**Figure S6. Aβ42 octameric β-sandwich oligomer model shows conductivity exclusively via β-sheet edges. A** Averaged contact frequencies for direct contacts between water, lipid phosphates and lipid carbonyl oxygens to protein atoms mapped onto the octameric β-sandwich structure (O-AP) shown in cartoon representation. Side-chain atoms for residues 12-20 are shown in stick representation (left). Top view of the structure (right) with residues L34 and M35 side-chain atoms in the β-sandwich interior are additionally highlighted. **B** Representative snapshot from different view points illustrates the specific interactions of the HHQK (residues 13-16) domain from the side-by-side β1-strand pair edges with individual POPC lipid molecules in the membrane center. **C** Stable edge pore in POPC bilayer formed by β-sandwich octameric oligomer shown in side and top view. Lipid phosphate groups are shown as spheres and colored according to their distance from the bilayer center.

**Figure S7. Aβ42 tetrameric and hexameric β-barrel oligomer models. A** Building blocks and scaffolds of tetrameric (left) and hexameric (right) β-barrel Aβ42 oligomers. Renderings of initial coordinates of both oligomer models are shown in cartoon representation. Structure templates (PDB ID: 5W4J - left, PDB ID: 3SGO - right) used to model the β-barrel and cylindrin structures are shown in white ribbons. **B** Final oligomer models embedded in a POPC bilayer shown in side and top view. Lipid phosphorus atoms are shown as orange spheres.

**Figure S8. Extent and characteristics of polar transmembrane defects induced by Aβ42 β-barrel and cylindrin oligomer models (AMBER simulations).** **A** Occurrence of a continuous polar defect across the POPC bilayer and **B** partial density profiles for water, polar lipid groups, potassium and chloride ions. Data are averaged over multiple independent AMBER simulations, shading indicates the standard error. As reference, profiles and distributions for an unperturbed lipid bilayer are also reported (broken lines). **C** Averaged contact frequencies to water and lipid oxygen atoms are shown as mapping onto the starting structures of tetrameric (left) and hexameric (right) β-barrel oligomer models.

**Figure S9. Extent and characteristics of polar transmembrane defects induced by Aβ42 β-barrel and cylindrin oligomer models (CHARMM simulations). A** Occurrence of a continuous polar defect across the POPC bilayer and **B** partial density profiles for water, polar lipid groups, potassium and chloride ions. Data are averaged over multiple independent CHARMM simulations, shading indicates the standard error. As reference, profiles and distributions for an unperturbed lipid bilayer are also reported (broken lines). **C** Averaged contact frequencies to water and lipid oxygen atoms are shown as mapping onto the starting structures of tetrameric (left) and hexameric (right) β-barrel oligomer models.

**Figure S10. Extent and characteristics of polar transmembrane defects induced by the Aβ42 O-AP model as a function of the K16 protonation state. A** Partial density profiles for water, polar lipid groups, potassium and chloride ions are compared for simulations with the protonation state of the titratable amino acid K16/LysH^+^ (positively charged, black) or K16/Lys^0^ (deprotonated form, blue) in AMBER (left) and CHARMM (right) force fields. **B** Occurrence of a continuous polar defect across the POPC bilayer. Data are averaged over multiple independent simulations, shading indicates the standard error. **C** Density profiles show location of the HHQK domain (residues 13-16) inside the POPC layer. Shading indicates the standard error.

**Figure S11. Aβ42 oligomer models with the 6RHY fold are stabilized by lower pH. A** The panel reports the average r.m.s.d. of the side-by-side β1-strand pair edge with respect to the simulation starting structure. Simulations of octa-, hexa-, and tetrameric Aβ42 β-sandwich models are compared with the protonation state of the titratable amino acid K16/LysH^+^ (positively charged, black) or K16/Lys^0^ (deprotonated form, blue) in AMBER and CHARMM force fields. **B** The average r.m.s.d. of the side-by-side β1-strand pair edge with respect to the simulation starting structure are shown as function of the average polar transmembrane defect.

**Figure S12. Polar transmembrane defects induced by smaller Aβ42 β-sandwich models with the 6RHY fold depend on force field. A** Tetrameric Aβ42 β-sandwich model two side-by-side β1-strand pair edges composed of β1- and β3-strands, respectively. Occurrence of a continuous polar defect across the POPC bilayer is shown for the tetrameric β-sandwich model in **B** AMBER and **C** CHARMM force fields. **D** Time traces show spontaneous formation of polar defects across the membrane, i.e. water and polar lipid groups entering into the hydrophobic membrane core induced by the tetramer Aβ42 oligomer model (black - AMBER; burgundy - CHARMM). The gray circle indicates the time point where CHARMM simulation were started from initial coordinates (t0) of pore-forming structures sampled from the AMBER simulations. **E** Data averaged over five independent AMBER to CHARMM simulations, shading indicates the standard error.

**Supplementary Figures**

**Figure S1**


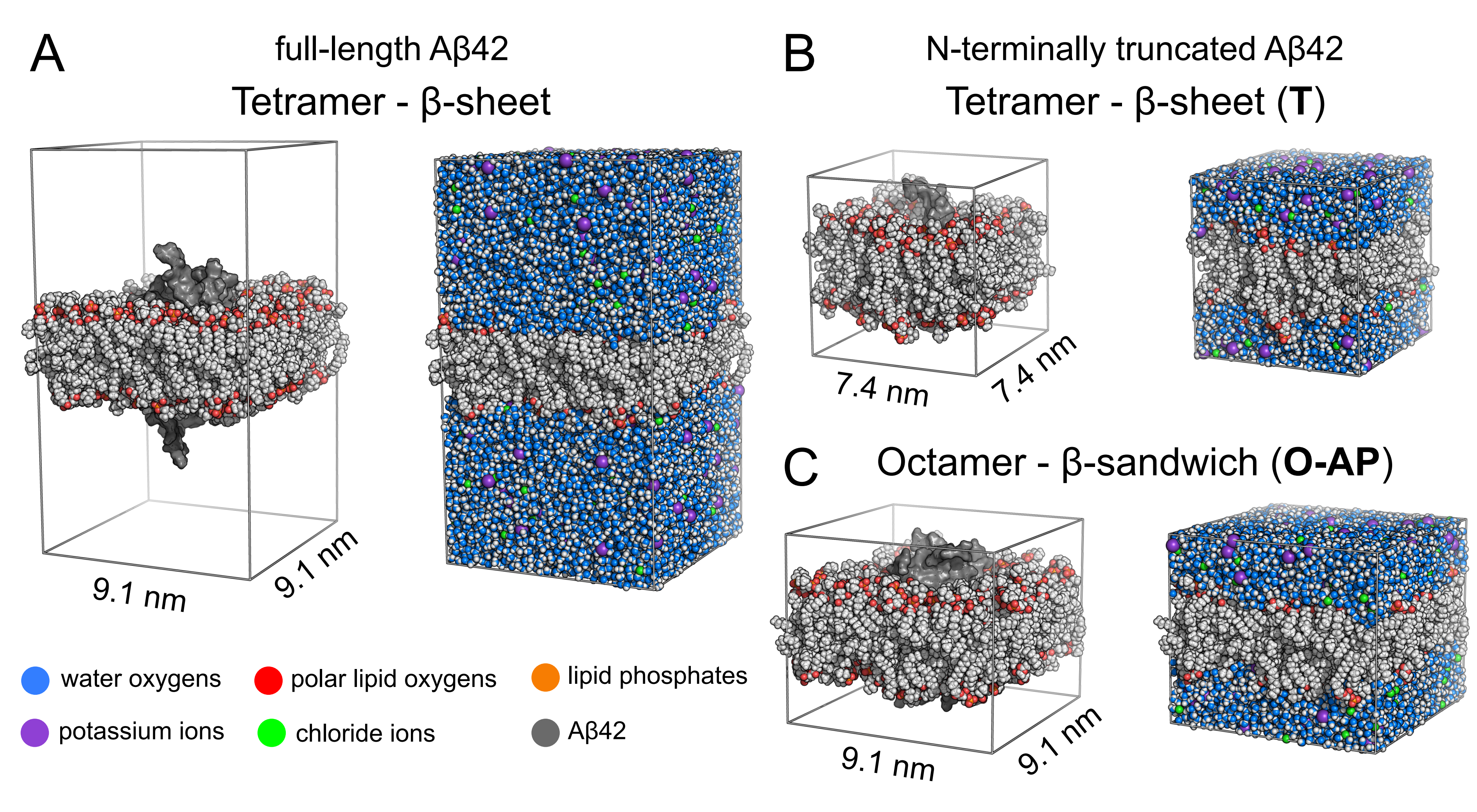


**Figure S2**

**
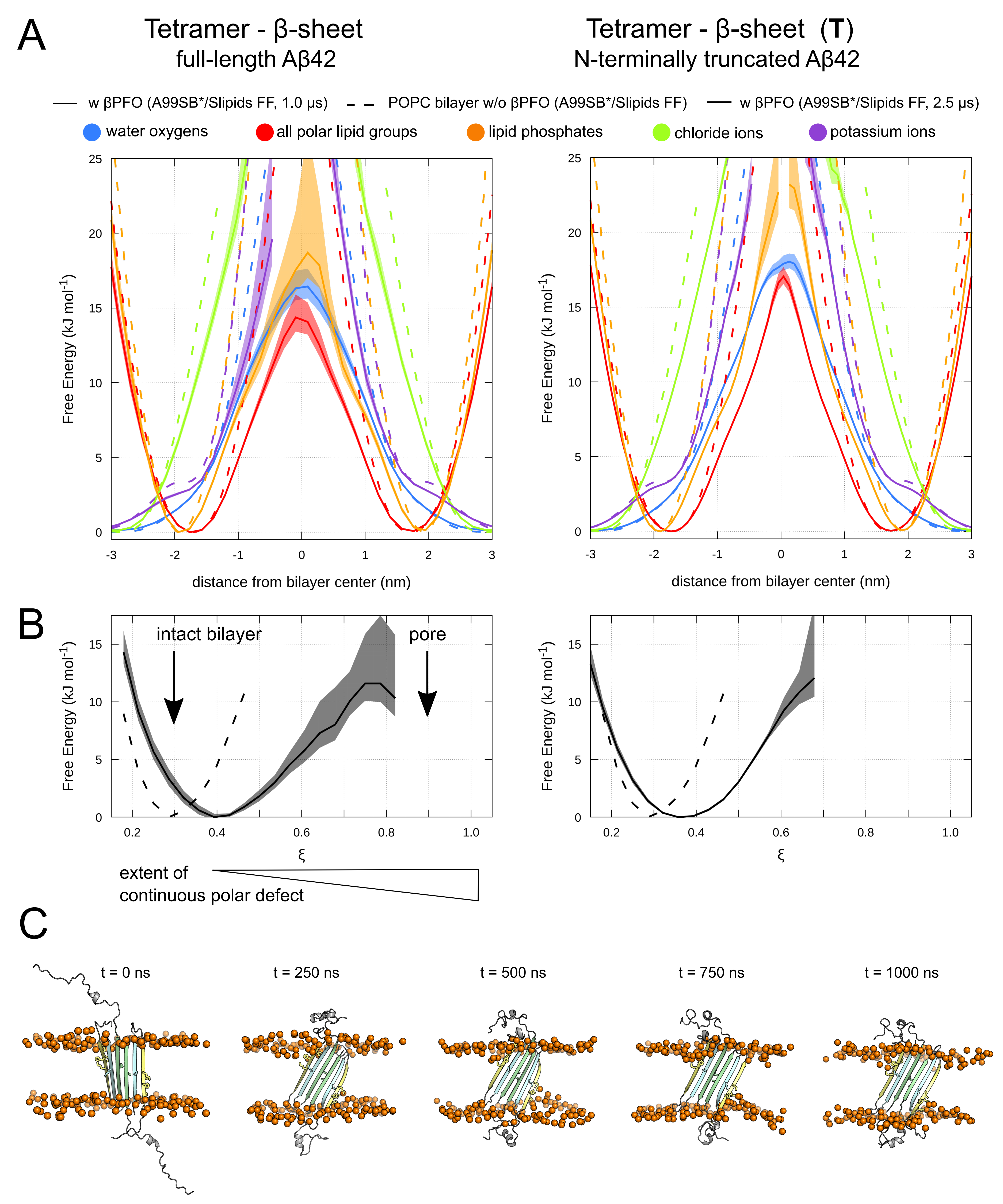
**

**Figure S3**

**
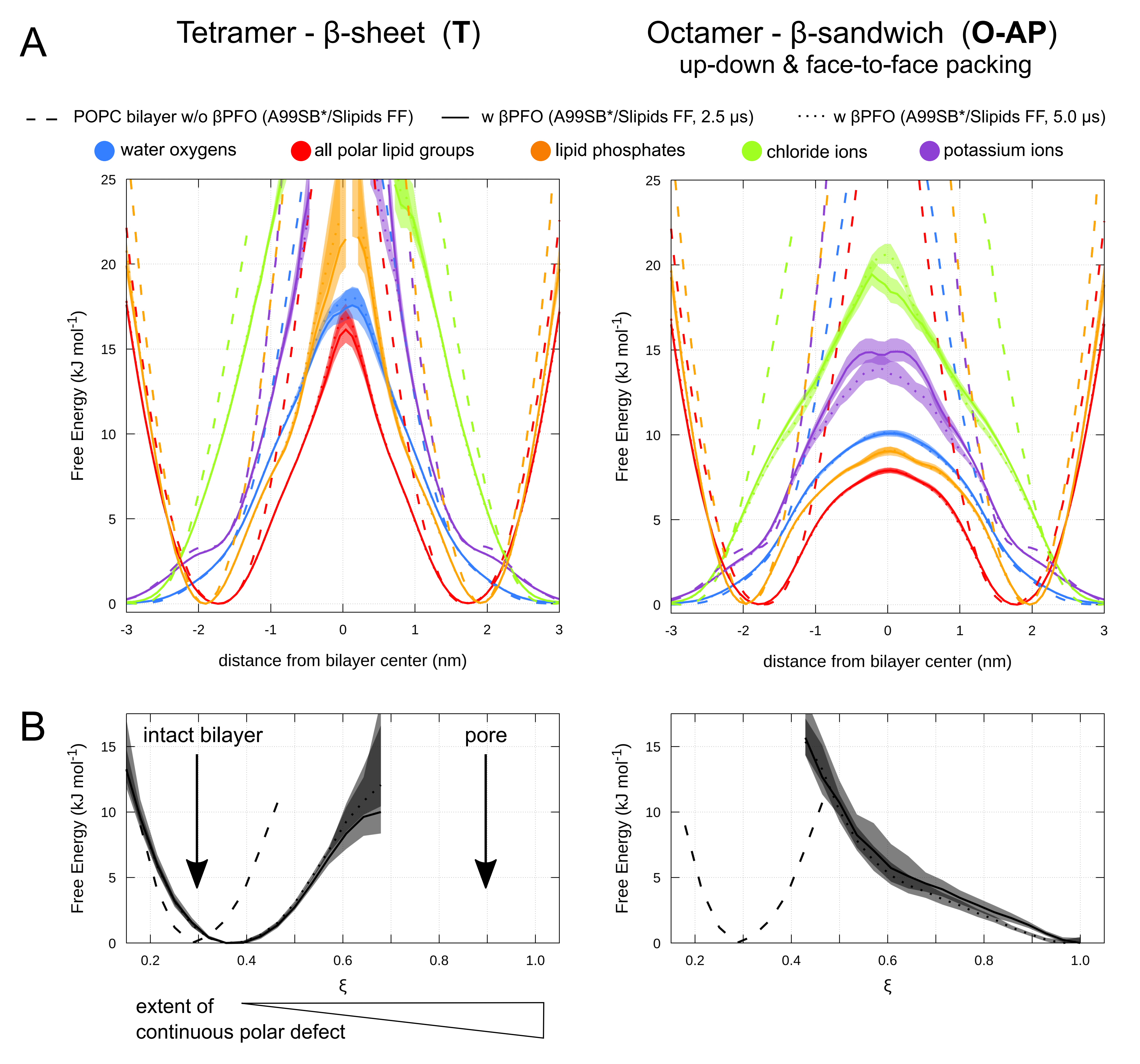
**

**Figure S4**

**
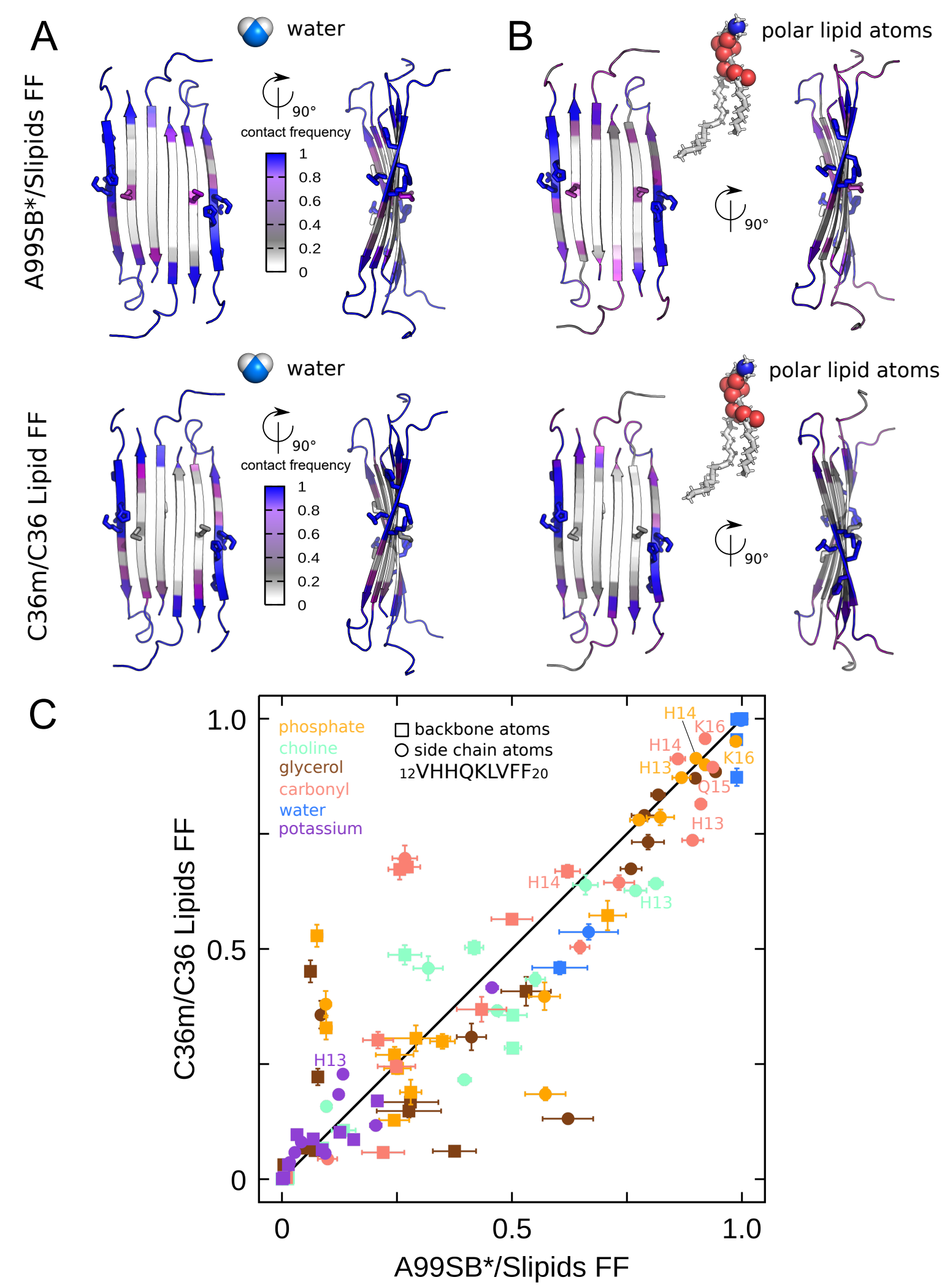
**

**Figure S5**

**
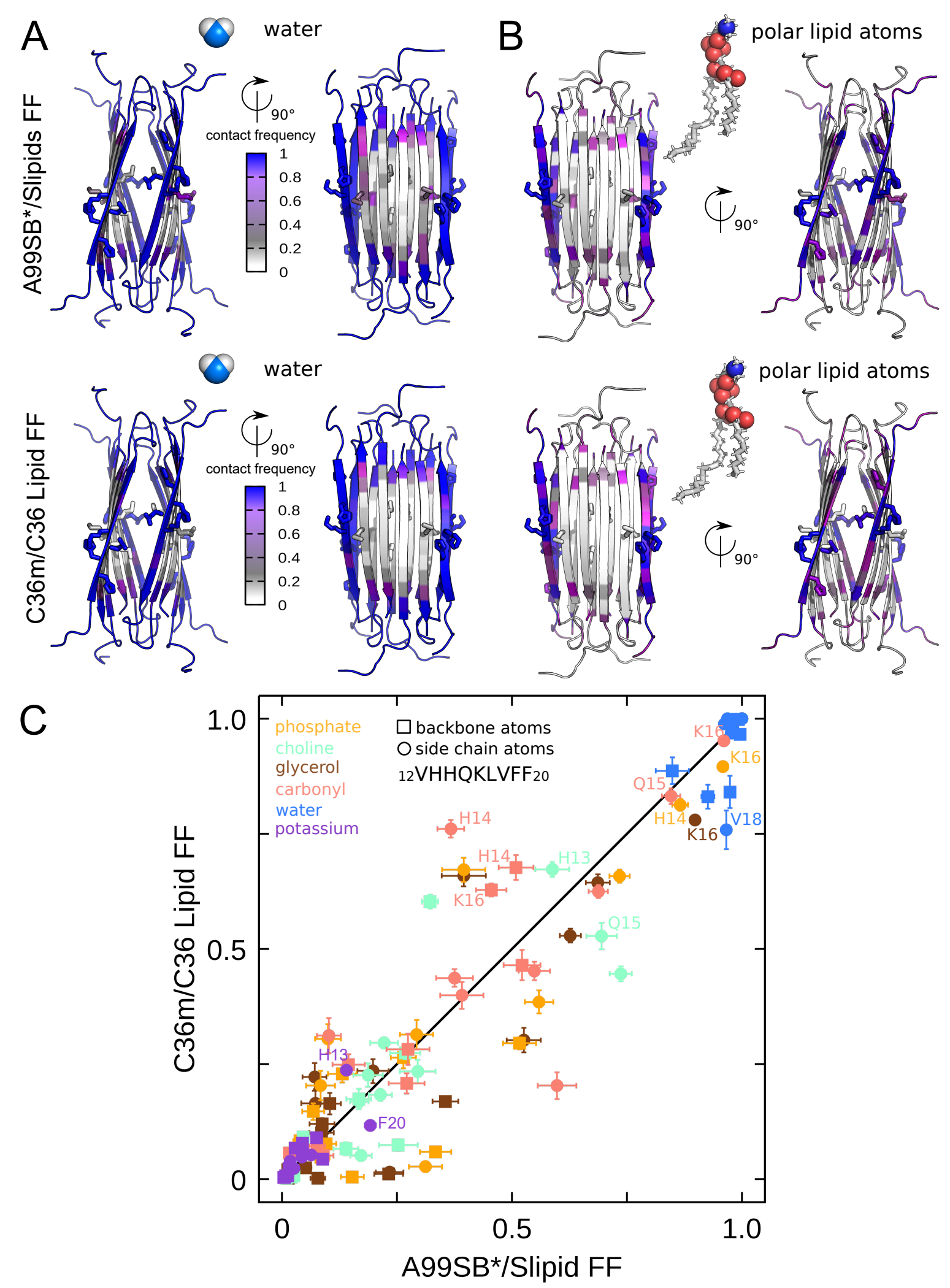
**

**Figure S6**

**
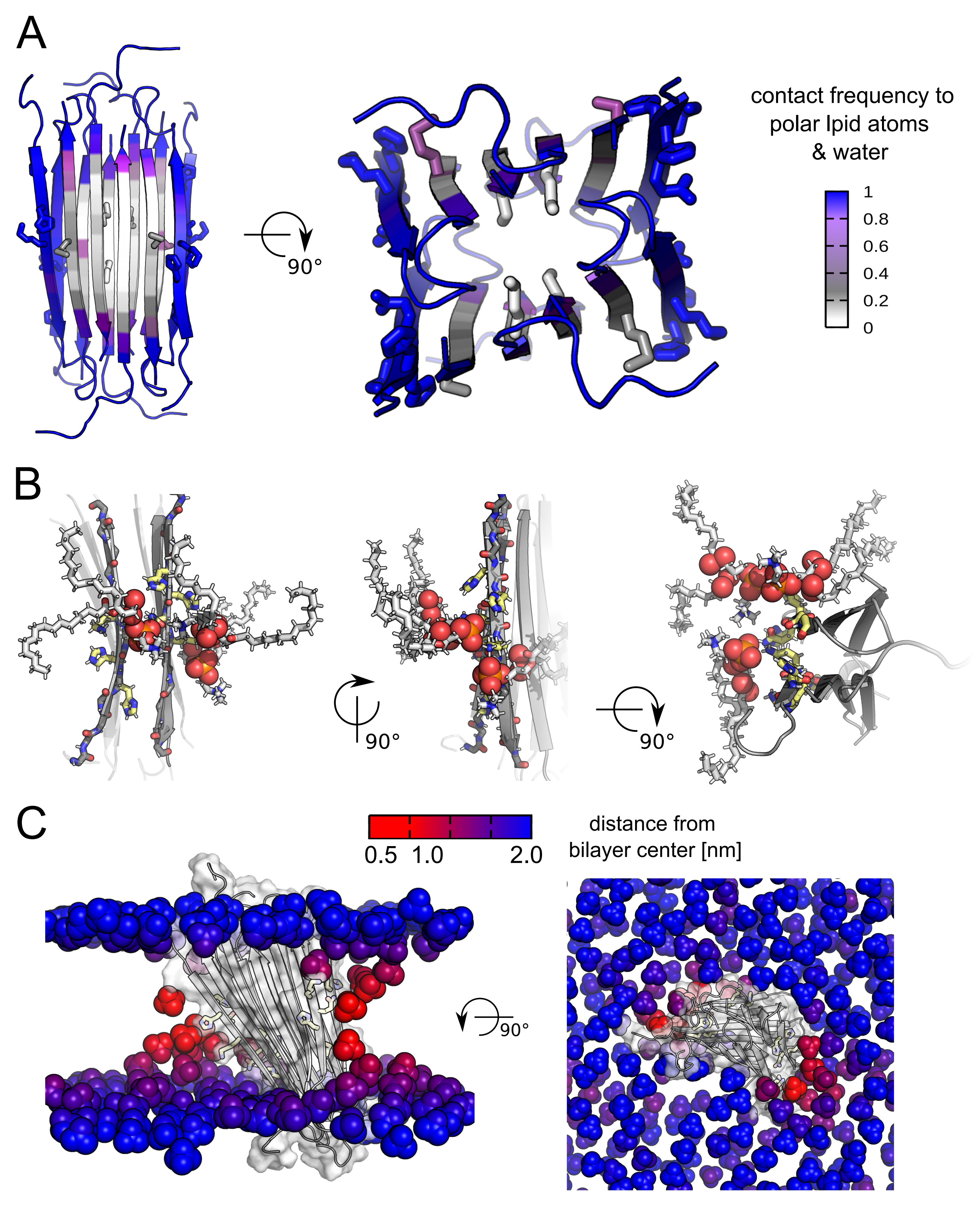
**

**Figure S7**

**
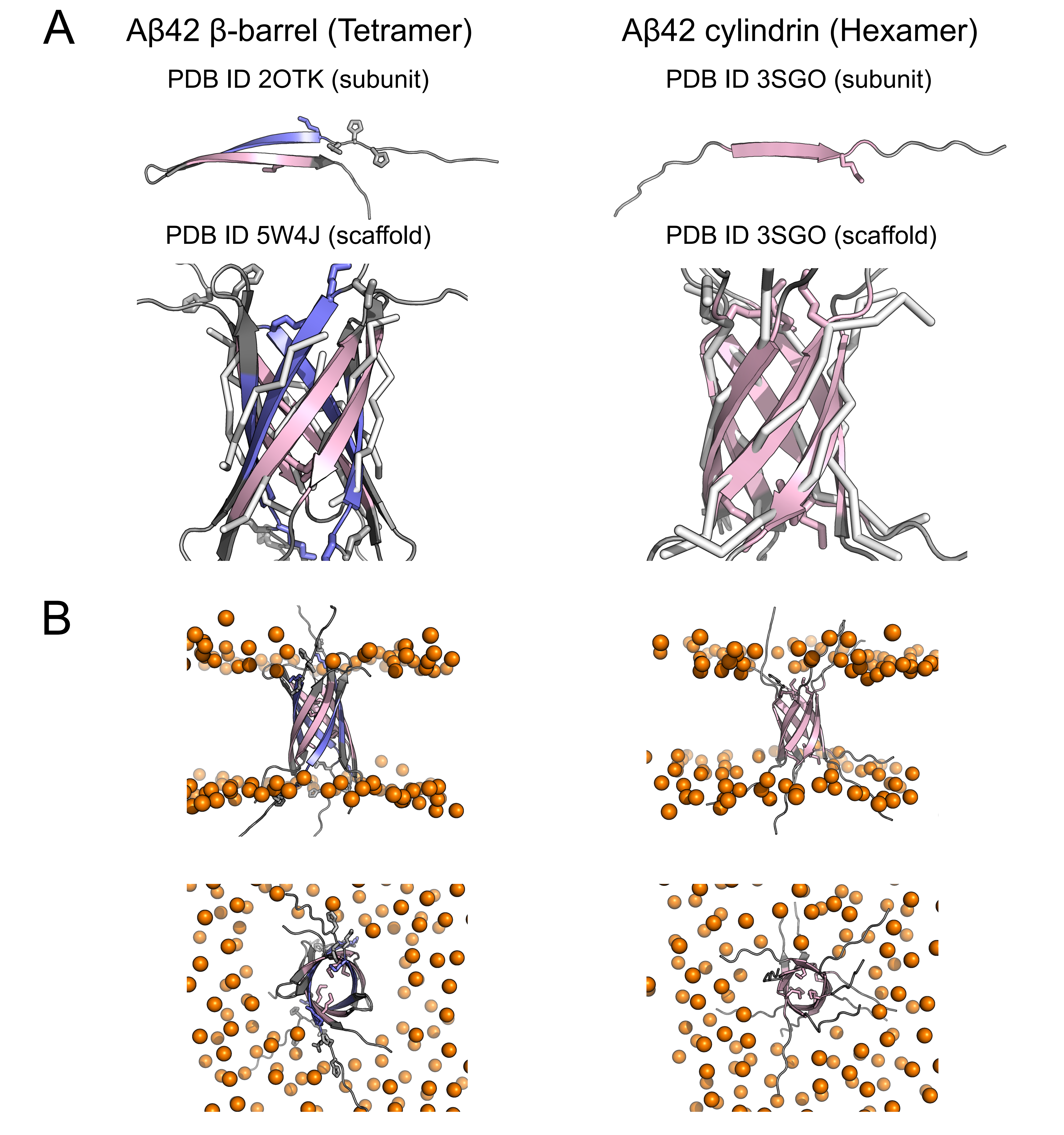
**

**Figure S8**

**
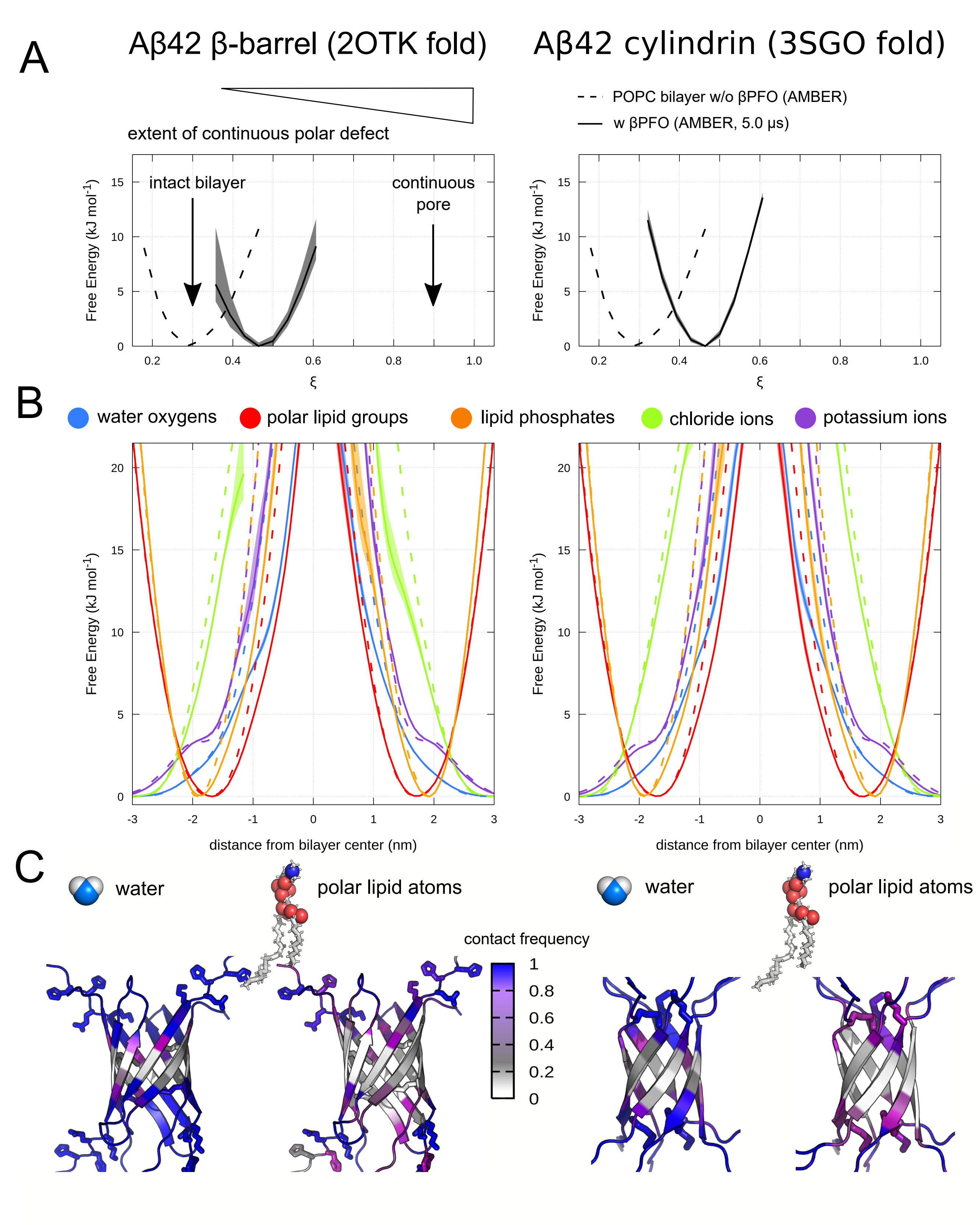
**

**Figure S9**

**
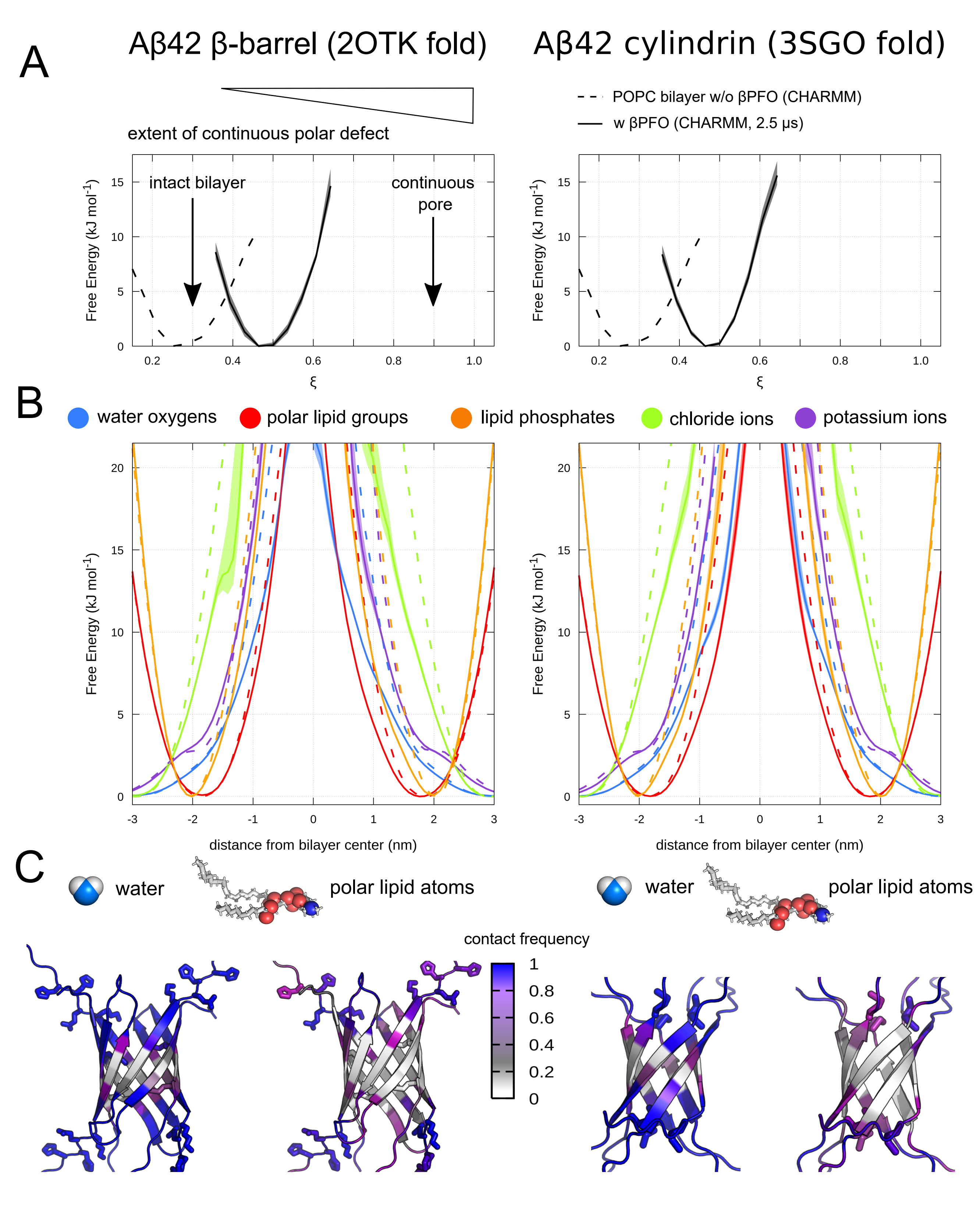
**

**Figure S10**

**
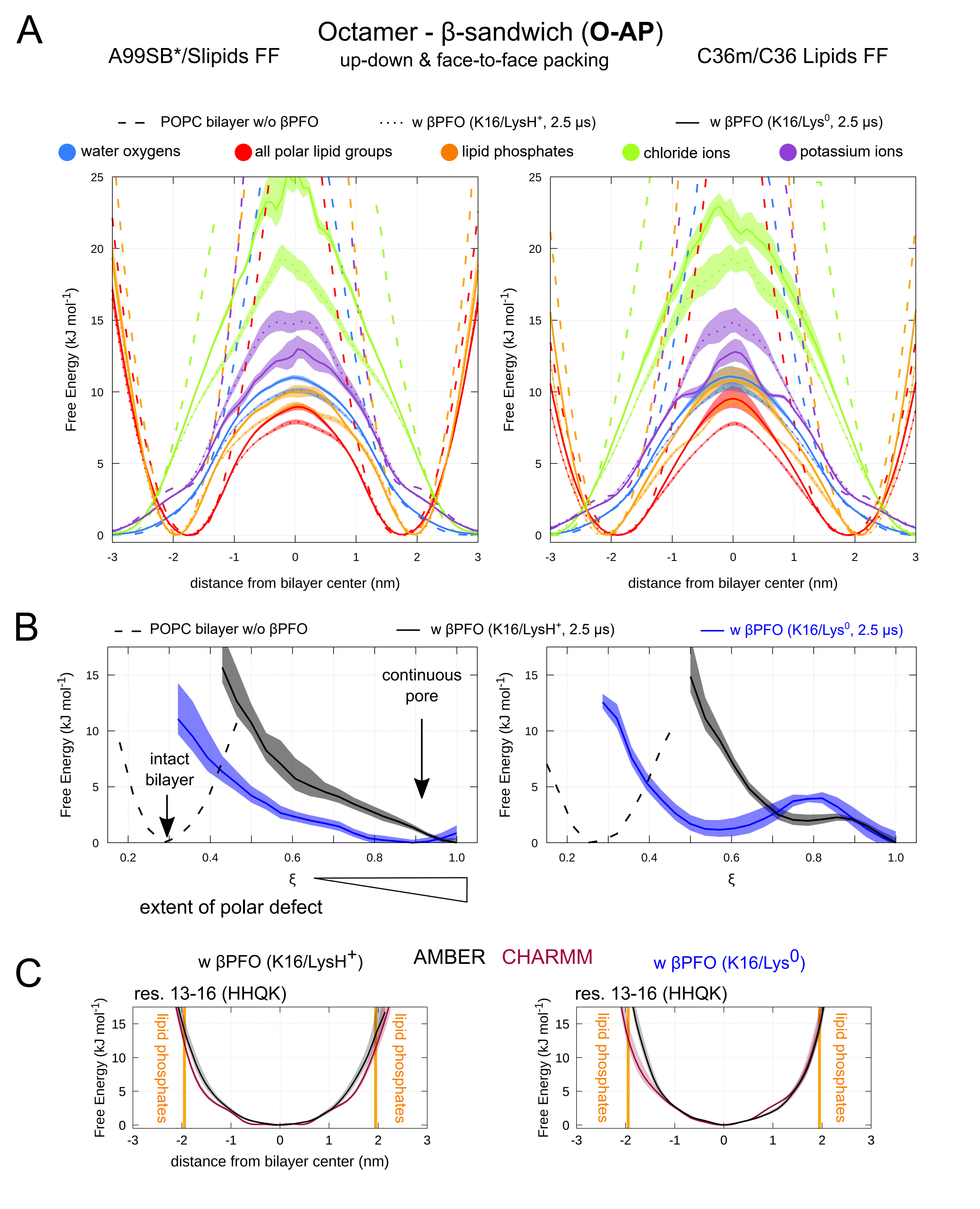
**

**Figure S11**

**
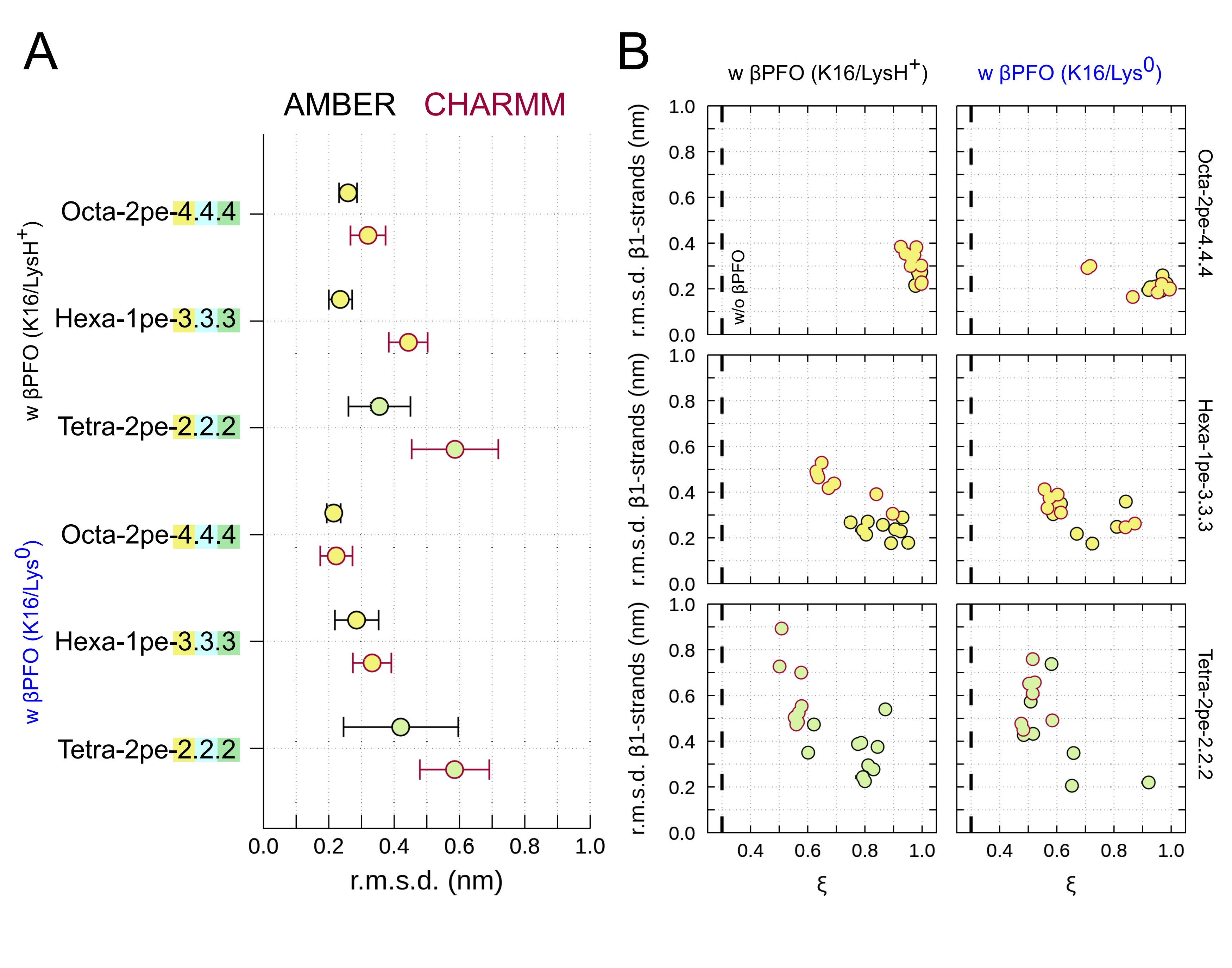
**

**Figure S12**


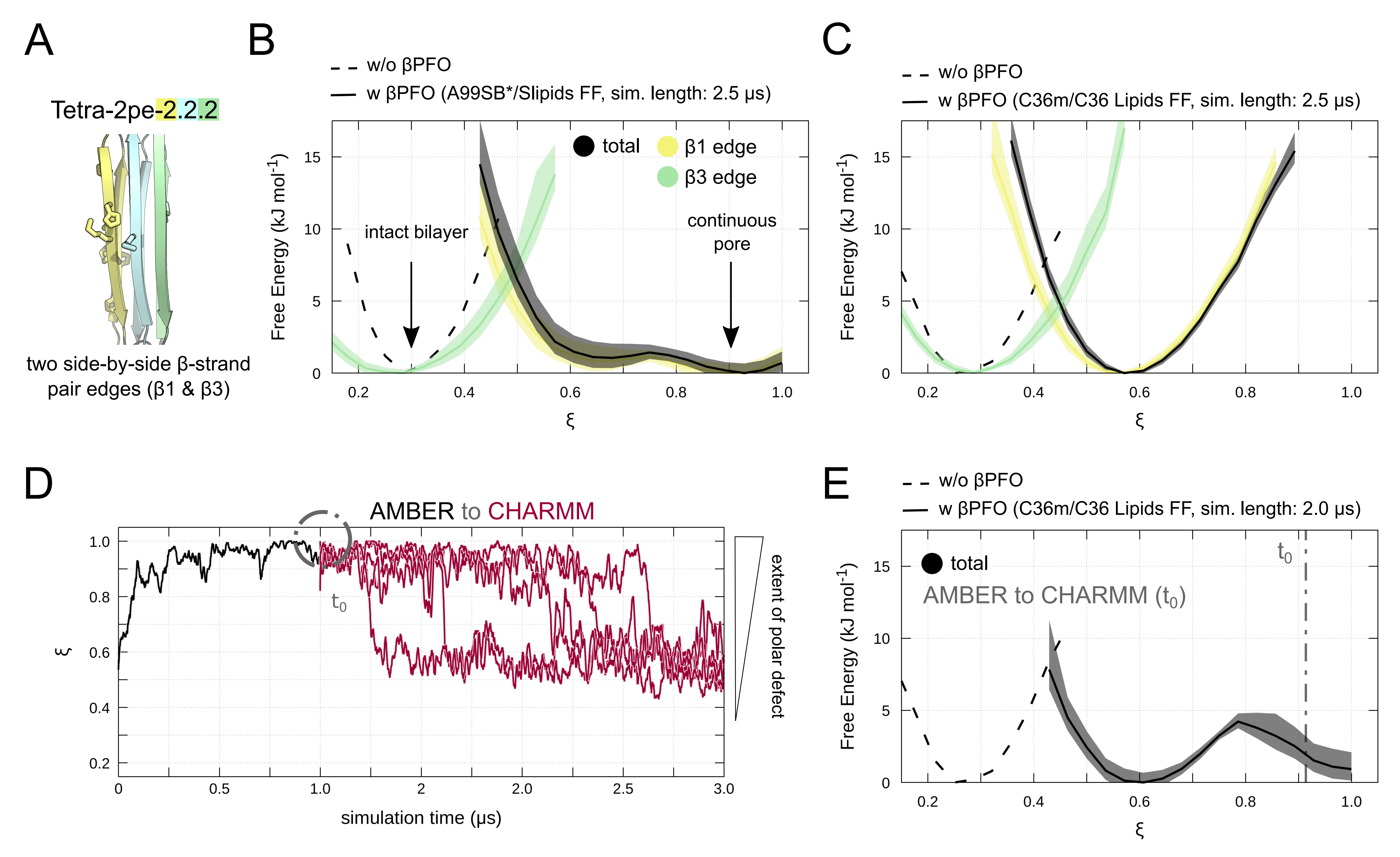

Supplement: Supplementary Tables and Figures [file mmc4.docx]
